# Genome-Wide Association Studies and Heritability Estimates of Body Mass Index Related Phenotypes in Bangladeshi Adults

**DOI:** 10.1371/journal.pone.0105062

**Published:** 2014-08-18

**Authors:** Molly Scannell Bryan, Maria Argos, Brandon Pierce, Lin Tong, Muhammad Rakibuz-Zaman, Alauddin Ahmed, Mahfuzar Rahman, Tariqul Islam, Muhammad Yunus, Faruque Parvez, Shantanu Roy, Farzana Jasmine, John A. Baron, Muhammad G. Kibriya, Habibul Ahsan

**Affiliations:** 1 Department of Health Studies, University of Chicago, Chicago, Illinois, United States of America; 2 UChicago Research Bangladesh, Dhaka, Bangladesh; 3 International Centre for Diarrhoeal Disease Research, Bangladesh, Dhaka, Bangladesh; 4 Department of Environmental Health Sciences, Columbia University, New York, New York, United States of America; 5 Department of Medicine, University of North Carolina, Chapel Hill, North Carolina, United States of America; Children's National Medical Center, Washington, United States of America

## Abstract

Many health outcomes are influenced by a person's body mass index, as well as by the trajectory of body mass index through a lifetime. Although previous research has established that body mass index related traits are influenced by genetics, the relationship between these traits and genetics has not been well characterized in people of South Asian ancestry. To begin to characterize this relationship, we analyzed the association between common genetic variation and five phenotypes related to body mass index in a population-based sample of 5,354 Bangladeshi adults. We discovered a significant association between SNV rs347313 (intron of NOS1AP) and change in body mass index in women over two years. In a linear mixed-model, the G allele was associated with an increase of 0.25 kg/m^2^ in body mass index over two years (p-value of 2.3·10^−8^). We also estimated the heritability of these phenotypes from our genotype data. We found significant estimates of heritability for all of the body mass index-related phenotypes. Our study evaluated the genetic determinants of body mass index related phenotypes for the first time in South Asians. The results suggest that these phenotypes are heritable and some of this heritability is driven by variation that differs from those previously reported. We also provide evidence that the genetic etiology of body mass index related traits may differ by ancestry, sex, and environment, and consequently that these factors should be considered when assessing the genetic determinants of the risk of body mass index-related disease.

## Introduction

Body mass index (BMI) can predict subsequent health outcomes. Both over- and underweight individuals increase their subsequent risk of death and poor health outcomes when compared to their normal-weight peers [1]–[6]. Similarly, a change in BMI over time is also associated with increased mortality and morbidity [7]–[11]. BMI has increased in low-income countries over the last three decades [12], which has increased the percentage of these populations who are now exposed to the risks associated with increased BMI. Despite this increase in average BMI, much of the population in Bangladesh and South Asia remains underweight (including 40% of our population-based sample). Underweight individuals in Bangladesh also show increased mortality [4], and studies in other Asian cohorts have demonstrated that low BMI is associated with increased mortality from a wide variety of causes, including cardiovascular disease, cancer, and respiratory disease [13], [14].

Although a person's BMI is affected by nutrition, genetics also exerts an influence on BMI-related phenotypes. This genetic component has been confirmed for point-in-time measurements of BMI related traits. Approximately 40 loci have been identified through genome-wide association studies (GWASs) [15]–[21], and heritability studies estimate that between 60%–80% of the phenotypic variance in BMI can be explained by genetic variation [22], [23]. Although these conclusions come from studies that were predominantly composed of populations of European descent, GWASs of participants with African [24], [25] and East Asian [26] ancestry suggest that associations between genetics and BMI-related traits can be found by studying a non-white population, such as our study sample.

The Bangladeshi participants in this study can provide novel insights into the genetic mechanisms of BMI-related traits. The participants of this study have a genetic architecture unique to South Asia, and were also exposed to widespread undernourishment (one quarter of our participants had a BMI lower than 17.6 kg/m^2^), both of which differentiates them from previously studied populations. Their genetics and nutritional habits create the conditions by which some of the genetic determinants of BMI related traits may differ from those previously identified. This study also investigates a phenotype, change in BMI, which has not been widely examined with GWASs or heritability estimates.

We conducted GWASs using genotyped and imputed data from a sample of Bangladeshi adults. We evaluated associations between genome-wide single nucleotide variants (SNVs) and the baseline phenotypes of BMI and height, as well as over- and underweight status. We also investigated whether there is evidence of a genetic component to the two-year change of BMI within the cohort. We repeated the GWAS analysis after we restricted the sample by sex and BMI classification at baseline, to investigate whether there was heterogeneity in the genetic determinants of BMI-related phenotypes. In addition, for each of the continuous phenotypes, we provided an estimate for the narrow sense (additive) heritability, as well as the phenotypic variability that is modeled by the additive effects of all measured and imputed SNVs.

This study will help to increase the knowledge of the genetic determinants of BMI-related traits. We also introduce a BMI-related trait that has previously not been investigated as heritable: change in BMI, and suggest that genetic variation may drive this phenotype. We characterize the variants that contribute to BMI-related traits for the first time in South Asians, and suggest that the genetic variants that determine BMI related traits may differ than the variants identified in previously studied populations, and differ by gender. These results will help further elucidate the differences and similarities among populations in terms of the etiology of BMI. This biological insight may suggest how to control BMI-related morbidity, how to classify patients by risk of developing BMI-related diseases, and suggest populations that may be helped by interventions that may stabilize a person's BMI.

## Materials and Methods

### Study Sample

The Health Effects of Arsenic Longitudinal Study (HEALS) [27] is a population-based cohort study established in 2000 in Araihazar, Bangladesh to primarily study the health effects of long-term arsenic exposure. The study enrolled a total of 20,033 participants in two recruitment cycles. The Bangladesh Vitamin E and Selenium Trial (BEST) [28] was established in Araihazar and surrounding areas in 2006 to determine whether 7,000 arsenic-exposed participants who supplemented their diet with vitamin E and selenium would reduce their rate of non-melanoma skin cancer. Clinical and anthropomorphic measurements were ascertained biennially by trained clinicians and interviewers, providing longitudinal information on the participants. Demographic and clinical characteristics of the study participants included in this analysis are shown in Table S1.

### Genotype Data

A total of 5,499 participants from HEALS and BEST were genotyped using the Illumina Infinium: HumanCytoSNP-12 v.2.1 chip. Details of the study sample selection, quality control, and genotyping process are reported elsewhere [29]. The participants were genotyped in two batches. After quality control, 5,354 out of 5,499 samples and 257,747 out of 299,140 SNVs were carried forward into imputation.

MaCH software [30] pre-phased the genotyped data, and minimac software [31] imputed the data, using the HapMap3 GIH reference panel. SNVs with a maximum likelihood r^2^ greater than 0.3 were used in analysis. A unix script assigned a hard call genotype probabilistically based on the minimac maximum likelihood probability. After imputation, 1,208,102 SNVs were used for association analyses.

### Assessment of Outcomes and Covariates

At baseline and biennial follow-up, study physicians measured a participant's weight and height. Weight was measured three times over the course of the interview with one of two Misaki scales that were manufactured in Japan (serial numbers: 67117, 58216). The scales were calibrated weekly. The average value of the three measurements was recorded. Height was measured by placing the scale on the participant's head, parallel to the floor, and measuring the length from the scale to the floor with a locally manufactured tape measure three times. The average value of the three measurements was recorded. BMI was calculated by dividing average weight in kilograms divided by the square of the average height in meters. Participants with a BMI less than 18.5 kg/m^2^ were classified as underweight, and participants with BMI greater than or equal to 23 kg/m^2^ were classified as overweight. A cutoff of 23 kg/m^2^ was chosen for classification of overweight status (as opposed to 25 kg/m^2^) based on evidence suggesting that people of Asian descent have higher adiposity and risk of obesity-related comorbidities at lower BMI than do people of European descent [32], [33]. Gender and age were ascertained during the interview.

### Exclusions

Among the 5,354 participants with GWAS data, baseline BMI was missing on 11 participants, baseline height was missing on 10 participants, 103 participants were lost to follow up after baseline, and an additional 263 participants were missing a BMI measurement at their first follow up. Participants with missing information on a phenotype were excluded from analysis for that phenotype.

### Statistical Methods

We analyzed five phenotypes: BMI, height, underweight and overweight (both were only compared to their normal weight peers), and change in BMI over two years. Previous studies suggest that the genetic underpinnings of BMI-related traits may differ by sex [35], so we also conducted subsample analyses on BMI and change in BMI after we stratified by sex. We also stratified by the participant's BMI at baseline (those who were underweight at baseline, those who were normal weight at baseline, and those who were overweight at baseline), and re-analyzed BMI and change in BMI for the participants in each category.

Our interviews with the participants established that many participants were known to be distantly related, and our analysis of relatedness indicated that more than 60% of the participants were genetically related to at least one other participant as a third cousin or closer. Therefore, we used the software Genome-wide Efficient Mixed-Model Association (GEMMA) [34] to control for this between-subject genetic correlation. This software calculated a relatedness matrix based on the pairwise covariance between genotypes, and then estimated the effect of each SNV on the phenotype while controlling for the relatedness matrix with a linear mixed model. We assumed an additive model of inheritance, and treated all phenotypes as continuous variables. We also controlled for the linear effects of baseline age, the square of baseline age, sex, and genotyping batch. The reported p-values are from a Wald test. We considered 5·10^−8^ to be the cutoff for genome wide significance. R software [35] and LocusZoom [36] were used to plot results, and ANNOVAR [37] was used to annotate the context and nearest genes of the variants.

We did not transform the continuous variables, as they are distributed roughly normally in this population, and the linear mixed model technique used by GEMMA is relatively robust to deviations from normality [38].

For the continuous traits, we estimated three aspects of heritability (described below) using Genome-wide Complex Trait Analysis software (GCTA) [39]. Similar to GEMMA, this software also estimates a relatedness matrix based on the pairwise genetic covariance. We first estimated h_g_^2^, the amount of variance in the trait that was explained by the interrogated SNVs. For h_g_^2^, GCTA fits the given phenotype with a linear mixed model, while it uses the estimated relatedness matrix as the variance term, to estimate the variance explained by all SNVs. We repeated the h_g_^2^ analysis after we restricted our sample to only one participant of any pair where the estimated kinship coefficient was larger than 0.025 (approximately 2^nd^-3^rd^ cousins), to reduce the possibility that the estimates would be inflated because of shared environment. We also estimated the full narrow sense heritability, h^2^, using the method described by Zaitlen et al [40]. This procedure is similar to the one described above, but replaces the full relatedness matrix with a modified one that assumes zero relatedness between participants whose estimated relatedness is less than 0.05. This modified relatedness matrix better approximates the identity by descent matrix.

### Ethics Statement

The study procedures and consent procedures were approved by the Columbia University Institutional Review Board and the Ethical Committee of the Bangladesh Medical Research Council. Since many of the people in rural Bangladesh are unable to read, each individual who agreed to participate provided verbal consent, which was recorded by the field staff physicians in the interview form in the presence of a witness. The study team explained details of the study procedures and the benefits and risks of the study in local language. Participants were advised that they could consent with or without donating blood or urine. The study team also explained to the participants that they could withdraw from the study at any stage, even if they had already provided consent, and explained the procedure for withdrawal.

## Results

### Genome-Wide Association Analyses: Baseline BMI and Height, and Baseline Over- or Under-weight Status

We carried out GWASs on four BMI-related phenotypes that were measured at the baseline visit: BMI, height, overweight status and underweight status. No SNVs were associated with any of the baseline traits with a p-value that reached the genome-wide threshold. Table 1 lists SNVs associated with all phenotypes for which we estimated a p-value of less than 10^−6^. Quantile-quantile plots suggest that the p-values for baseline BMI are enriched for small p-values, compared to the null hypothesis of no genetic association (Figure S1a).

**Table 1:** Top Associations between SNVs and BMI-Related Traits.

Phenotype	n analyzed	Chr	SNV	position	context	nearest gene (s)	RA	AA	RAF	β	se	p-value
**BMI**	5,343	5	rs955423	165652228	intergenic	TENM2	A	C	0.610	0.335	0.066	3.45·10^−7^
**BMI**	5,343	4	rs3096490	110198580	intronic	COL25A1	G	A	0.529	0.317	0.063	5.32·10^−7^
**BMI**	5,343	5	rs1392280	165608261	intergenic	TENM2	G	A	0.659	0.332	0.067	6.90·10^−7^
**BMI**	5,343	15	rs1426654	46213776	exonic	SLC24A5	A	G	0.510	0.325	0.066	7.54·10^−7^
**BMI**	5,343	4	rs794149	110179691	intronic	COL25A1	A	G	0.521	0.314	0.064	8.36·10^−7^
**BMI**	5,343	4	rs3096483	110189985	intronic	COL25A1	T	C	0.524	0.313	0.063	8.37·10^−7^
**Change in BMI**	4,983	5	rs10041997	120226210	intergenic	PRR16,FTMT	A	G	0.518	0.138	0.027	4.40·10^−7^
**Change in BMI**	4,983	5	rs1347155	171901953	intergenic	SH3PXD2B,NEURL1B	T	C	0.042	0.341	0.068	4.97·10^−7^
**Change in BMI**	4,983	2	rs7565158	213302215	intergenic	ERBB4,MIR4776-2	A	C	0.495	0.136	0.027	6.34·10^−7^
**Height**	5,344	15	rs8042988	87213283	intronic	ACAN	A	G	0.621	0.006	0.001	3.81·10^−7^
**Height**	5,344	14	rs4898878	55462859	intergenic	LINC00520,PELI2	A	G	0.709	0.006	0.001	9.07·10^−7^
**Underweight**	4,474	14	rs12882679	78916991	intronic	NRXN3	G	A	0.687	0.057	0.012	9.57·10^−7^
**Overweight**	3,244	14	rs1475010	25206451	intergenic	STXBP6,NOVA1	C	A	0.307	0.060	0.012	5.74·10^−7^
**Overweight**	3,244	14	rs8181969	25214651	intergenic	STXBP6,NOVA1	C	A	0.305	0.059	0.012	8.49·10^−7^

AA: Alternative Allele; the allele associated with a decrease in the phenotype; BMI: Body Mass Index; Chr.: Chromosome; Overweight: BMI ≥23 kg/m^2^; compared to normal weight; Position: From build 36.1; RA: Risk allele; the allele associated with an increase in the phenotype; RAF: Risk Allele Frequency; SNV: Single Nucleotide Variant; se: Standard Error; Underweight: BMI <18.5 kg/m^2^, compared to normal weight.

**Figure 1:**
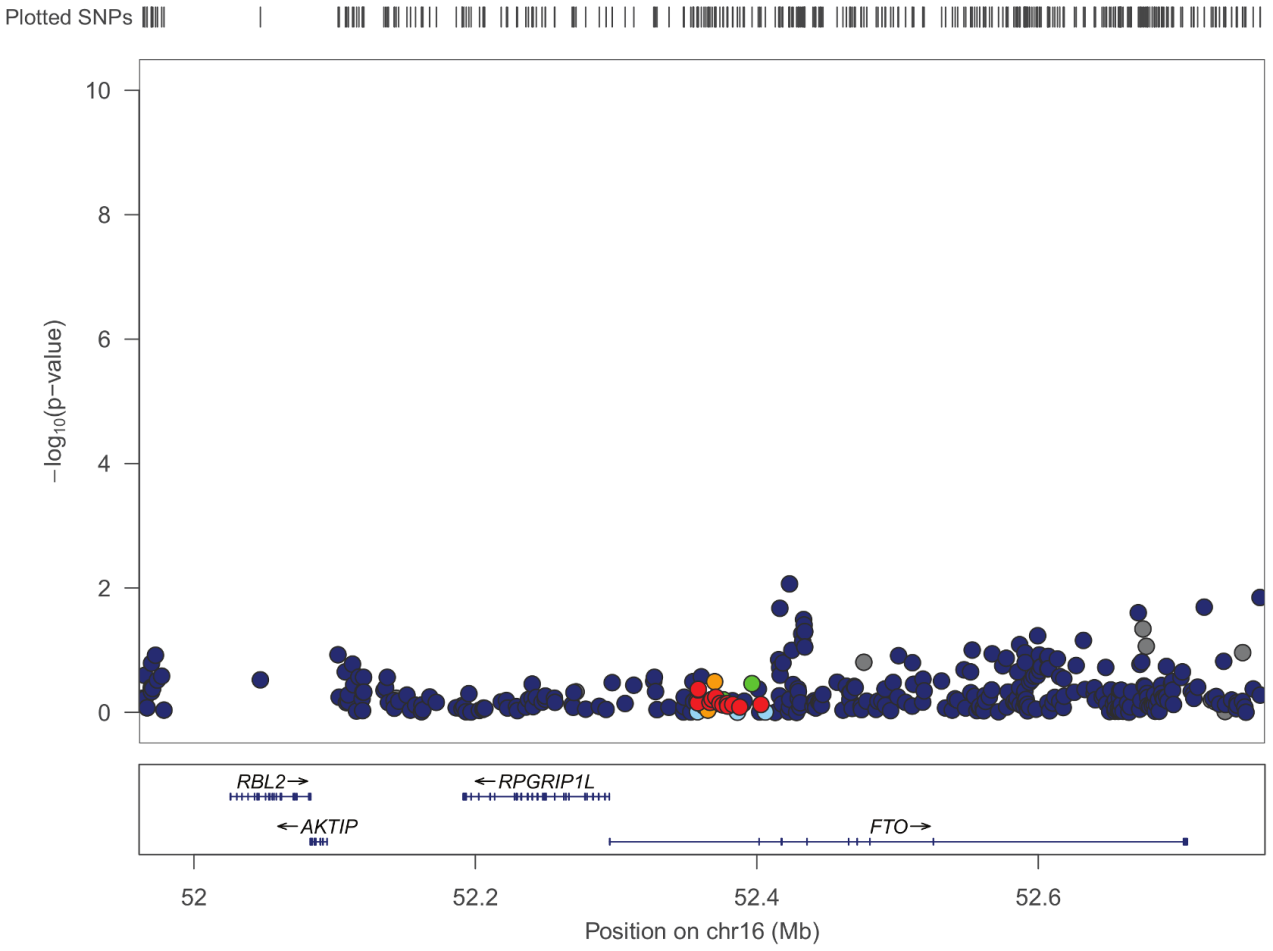
P-values for Baseline BMI in the Vicinity of the FTO Gene. p-values in the vicinity of the FTO gene indicate no obvious association with body mass index near the gene.

Ten previously published GWASs investigated cross-sectional BMI and reported a genome-wide significant association for a variant within or around the fat mass and obesity-associated protein (FTO) gene [13]–[15], [21], [22], [36]–[40]. Visual inspection of our data does not show convincing evidence for a strong signal in the FTO region (Figure 1).

**Figure 2:**
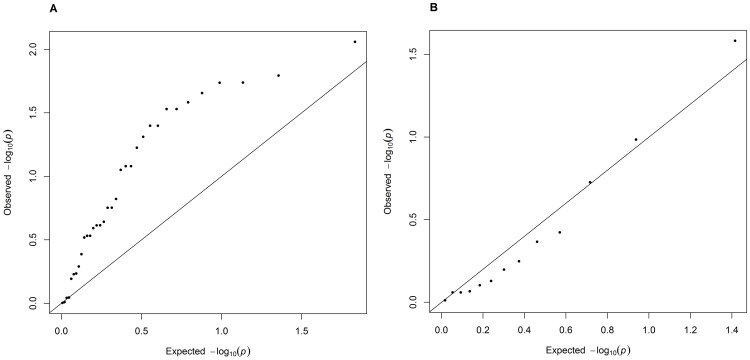
QQ Plots for SNVs Previously Associated with Baseline BMI. QQ plots for the 47 SNVs previously reported as associated with body mass index that we interrogated (A) SNVs that have a directionally consistent effect with previous literature (n = 35) show an inflation in the p-values calculated for this study, whereas the QQ plot of our p-values for SNVs that are directionally inconsistent (B) (n = 13) are consistent with the null hypothesis.

Our BMI GWAS interrogated forty-seven SNVs that the National Human Genome Research Institute (NHGRI) GWAS catalogue lists as associated with BMI with a p-value smaller than 10^−7^. Thirty-four of these overlapping SNVs (72%) have an estimated effect size in our analysis that is directionally consistent with previous reports. While we estimated a nominally significant (p<0.05) p-value for only 9 of these overlapping SNVs, the SNVs whose estimated effects are in the same direction as previous literature have significance tests that are enriched for small p-values (Figure 2A). There is no such enrichment in the quantile-quantile plot of the SNVs where our reported effect direction is different than the reported literature (Figure 2B).

We next stratified the sample by sex and re-analyzed the GWAS. No SNVs reached the genome-wide threshold (Table 2). Quantile-quantile plots of the stratified analysis suggest an inflation of small p-values for baseline BMI for females (Figure S2b), and baseline overweight status for males (Figure S2g).

**Table 2:** Top Associations between SNVs and BMI-Related Traits, by Sex.

Phenotype	n analyzed	Chr	SNV	position	context	nearest gene (s)	RA	AA	RAF	β	se	p-value
BMI: Male	2,590	No variants were associated with a p-value less than 10^−6^										
BMI: Female	2,753	15	rs1426654	46213776	exonic	SLC24A5	A	G	0.511	0.484	0.095	3.74·10^−7^
Height: Male	2,590	No variants were associated with a p-value less than 10^−6^										
Height: Female	2,754	9	rs1331623	121239024	intergenic	DBC1,MIR147A	A	T	0.337	0.008	0.002	8.45·10^−7^
Underweight: Male	2,252	4	rs6833159	28441886	intergenic	MIR4275,PCDH7	T	C	0.735	0.087	0.017	2.99·10^−7^
Underweight: Female	2,222	No variants were associated with a p-value less than 10^−6^										
Overweight: Male	1,444	15	rs12101726	92382946	intergenic	RGMA,MCTP2	C	A	0.195	0.101	0.019	2.42·10^−7^
Overweight: Male	1,444	6	rs1161397	50704412	intergenic	DEFB112,TFAP2D	A	G	0.105	0.127	0.026	9.45·10^−7^
Overweight: Male	1,444	6	rs280325	50743465	intergenic	DEFB112,TFAP2D	G	A	0.100	0.131	0.027	9.89·10^−7^
Overweight: Female	1,800	No variants were associated with a p-value less than 10^−6^										
Change in BMI: Male	2,340	4	rs6447650	40075967	intergenic	CHRNA9,RBM47	T	C	0.044	0.416	0.080	2.39·10^−7^
Change in BMI: Female	2,643	1	rs347313	160570900	intronic	NOS1AP	G	A	0.322	0.249	0.044	2.32·10^−8^
Change in BMI: Female	2,643	1	rs347306	160569259	intronic	NOS1AP	G	A	0.324	0.241	0.044	5.37·10^−8^
Change in BMI: Female	2,643	5	rs1347155	171901953	intergenic	SH3PXD2B,NEURL1B	T	C	0.042	0.559	0.104	9.34·10^−8^
Change in BMI: Female	2,643	1	rs347282	160579377	intronic	NOS1AP	G	T	0.319	0.222	0.045	7.20·10^−7^

AA: Alternative Allele; the allele associated with a decrease in the phenotype; BMI: Body Mass Index; Chr.: Chromosome; Overweight: BMI ≥23 kg/m^2^; compared to normal weight; Position: From build 36.1; RA: Risk allele; the allele associated with an increase in the phenotype; RAF: Risk Allele Frequency; SNV: Single Nucleotide Variant; se: Standard Error; Underweight: BMI <18.5 kg/m^2^, compared to normal weight.

Lastly, we examined whether the association between common variation and BMI differed by baseline BMI category. In these restricted GWAS, the analysis of the 869 participants who were overweight at baseline identified three SNVs that were genome-wide significant, and eight more that were suggestive of genome wide significance (p<10^−6^) (Table S2, Figure S2c). However, after closer examination, the participant in our study with the largest measured BMI carried the risk allele at each of these loci. Even though the BMI measurement for this participant seemed to be accurate, we were concerned that this one observation was driving the observed association (this participant's BMI was 51 kg/m^2^, and the participant with the second largest BMI measured 37 kg/m^2^). When we repeated the analysis excluding this one participant, no SNV rose to the level of genome-wide significance (Table S2, Figure S3d).

### Genome-Wide Association Analysis: Change in BMI

On average, our study participants increased their BMI 0.204 kg/m^2^ (confidence interval: 0.17–0.24 kg/m^2^) between their initial interview and their first follow up two years later. At baseline, increasing age was associated with a small decrease in BMI (Pearson correlation coefficient: -0.063, p-value: <0.0001), which suggested that the within-person increase was not a result of age-related weight gain.

We assessed with a genome-wide association study whether genetic variation affected a person's propensity to change their BMI over time. When examining the entire sample, no SNV was associated with this change in BMI phenotype with a p-value that reached the level of genome wide significance, although three of the SNVs were suggestive of association (Table 1).

**Figure 3:**
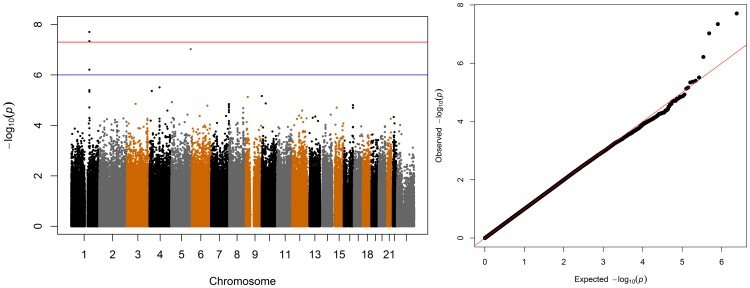
Manhattan and QQ Plots of Change in BMI Over 2 Years. A signal at chromosome 1, and inflation in the tails of the QQ plot indicate an association between SNV rs347313 and change in BMI over 2 years in females. This SNV is in the intron of NOS1AP. The suggestive threshold is drawn at 10^−6^, and the genome wide significance threshold is drawn at 5·10^−8^.

**Figure 4:**
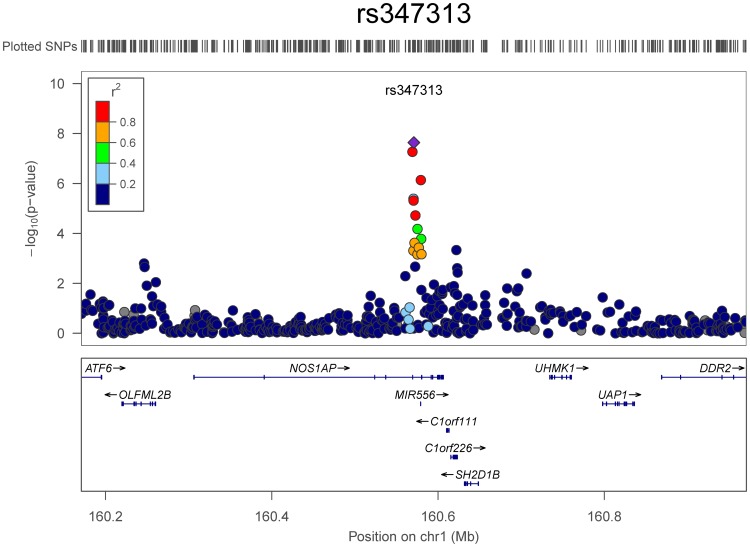
P-values for Change in BMI Over 2 Years in the Vicinity of NOS1AP. The association between SNV and change in BMI in females shows SNVs in linkage disequilibrium with rs347313 have inflated p-values.

As with the cross sectional BMI-related phenotypes, we investigated whether the SNVs that drove the change in BMI differed by sex, and found evidence that they do. The genotyped SNV rs347313 on chromosome 1 was significantly associated with two-year change in BMI at the genome-wide level (p = 2.3·10^−8^) (Figure 3). This G/A SNV is in the intron of the gene nitric oxide synthase 1 adaptor protein (NOS1AP) (Figure 4). The risk allele, G, was associated with a per-allele increase of 0.25 kg/m^2^ in BMI over the two-year period. This SNV was not associated with the cross-sectional measurements of baseline BMI, baseline weight, baseline height, or being over- or underweight at baseline in our analyses, and was also not associated with change in BMI for males (p>0.05 for all associations).

As with the cross-sectional BMI phenotypes, we also investigated whether the SNVs that are associated with a change in BMI differed by baseline weight, and found no evidence that they did (Table S2, Figure S3).

### Heritability

We estimated heritability for each of the three continuous traits in three ways: we used two methods to estimate h_g_^2^, which is the upper limit of the amount of phenotypic variance we could have expected to explain with our GWAS; and we estimated h^2^, the full narrow sense heritability.

We found evidence that the SNVs interrogated by our genotyping and imputation were associated with the phenotypic variation in the continuous BMI-related traits of this study population (Table 3, h_g_^2^ estimates). We repeated the h_g_^2^analysis, including one participant of any pair where the estimated kinship coefficient was larger than 0.025 to remove inflation in the h_g_^2^estimates that could be a result of phenotypic similarity due to shared environment. While this analysis reduced our sample size by half, the estimates were consistent with our full-sample h_g_^2^, albeit with wider standard errors.

**Table 3:** Heritability Estimates for Continuous BMI-related Traits.

		Using Whole Population					Excluding related >0.025		
Phenotype		n analyzed	Estimate of h^2 (a)^	se	Estimate of h_g_^2 (b)^	se	n analyzed	Estimate of h_g_^2 (c)^	se
**BMI**		**5,343**	**0.833**	**0.056**	**0.619**	**0.047**	**2,125**	**0.466**	**0.165**
	Male	2,590	1.000	0.076	0.792	0.077	751	0.943	0.462
	Female	2,753	0.653	0.141	0.588	0.097	1,374	0.602	0.259
**Height**		**5,344**	**0.634**	**0.064**	**0.460**	**0.050**	**2,125**	**0.352**	**0.173**
	Male	2,590	1.000	0.059	0.901	0.071	751	0.786	0.459
	Female	2,754	0.917	0.136	0.541	0.103	1,374	0.428	0.268
**Change in BMI**		**4,983**	**0.142**	**0.077**	**0.141**	**0.053**	**1,981**	**0.042**	**0.176**
	Male	2,340	0.312	0.114	0.263	0.091	666	0.169	0.483
	Female	2,643	0.149	0.159	0.086	0.102	1,315	0.000	0.265

se: standard error

(a) estimate of the full narrow sense heritability, calculated using GCTA, but replacing the full related matrix with a modified one that assumes zero relatedness between participants whose estimated relatedness is less than 0.05

(b) estimate of the amount of variance in the trait that was explained by the interrogated SNVs in a linear model, using the relatedness matrix calculated by GCTA, and using all participants

(c) estimate of the amount of variance in the trait that was explained by the interrogated SNVs in a linear model, using the relatedness matrix calculated by GCTA, and including only one participant of any pair where the estimated kinship coefficient was larger than 0.025 (2nd-3rd cousins).

We estimated the full narrow sense heritability using the method described by Zaitlen et al (Table 3, h^2^ estimates). The heritability estimates from that method are strikingly high, suggesting that the narrow sense heritability is dominated by shared environment, epistatic interactions, or dominance effects, and that this method might not be an appropriate estimate of the narrow sense heritability in our population.

As with the GWAS analyses, we repeated the heritability analysis, separating the participants by sex (Table 3). For all traits, men tended to have more phenotypic variance explained by the SNVs, although the estimates were imprecise. We also repeated the analysis, separating the participants by their baseline BMI categorization (Table S3).

## Discussion

In this analysis, we observed suggestive associations between several SNVs and BMI-related phenotypes measured at the baseline of our study. This begins to characterize the genetic determinants of BMI-related phenotypes for the first time in a South Asian population, although most associations did not reach genome-wide significance, and multiple phenotypes were tested. For some of the traits, the quantile-quantile plots are inflated, and the heritability estimates for the continuous phenotypes are significant, which suggests that a larger sample size may be able to detect SNVs with modest effect.

The results of our stratified analysis also suggest that the genetic drivers of BMI-related traits may vary by sex, and BMI categorization. For each of the sub-analyses, the SNVs that our stratified analyses identified as suggestive (p<10^−6^) did not overlap with the SNVs identified as suggestive in the pooled analysis. Since in the Bangladeshi population, both over- and underweight individuals increase their risk of morbidity from both communicable and non-communicable diseases, these results may help to develop risk scores that identify segments of a population that may be most susceptible to illness, allowing for more targeted intervention of anti-obesity and anti-malnourishment therapies.

These findings will be informative about which genetic determinants of BMI are consistent across populations, and which differ. It is compelling that our investigations show that many of the loci previously indicated as being associated with BMI [15], [17]–[20], [41], [42] contain no SNVs that are strongly associated with BMI in our sample. While the participants of these studies were primarily of recent European descent, one study in an African American population [21] and one in a Japanese population [22] found an association signal from a variant in the vicinity of FTO, which replicated a signal seen in populations of European ancestry [15], [18], [19], [41], [42]. This suggests that genetic variation at this locus affects BMI in people of differing backgrounds. These differences between the previous research and our study may be a result of the mechanisms by which genetics influences BMI. There is growing evidence that many of the variants identified in previous BMI GWASs affect BMI by influencing food choices or appetite [19], [43], [44]. However, in the rural Bangladeshi population, decisions about food may be driven less by preference, and more by availability and affordability, limiting the ability of any SNV that modifies behavior to affect BMI.

This analysis also reports the first genetic variation significantly associated with change in BMI that was discovered by interrogating the whole genome. The change in BMI for women with the risk allele is modest, 0.25 kg/m^2^ over two years, but weight loss as low as 0.2 kg/year, and gains as little as 0.6 kg/year have been found to be associated with all-cause mortality in a different population [45]. To our knowledge only one previous GWAS investigated adult change in BMI; it reported a null result [15]. The results presented here represent a much denser array of SNVs, and also have benefited from improvements in analysis since that study was published, such as control for population substructure, and imputation of non-genotyped SNVs. As this is one of the first studies to examine change in BMI in a genome-wide way, these findings need to be replicated. If the signal is replicated, further research needs to examine whether this same variant is also responsible for a genetic contribution to change in BMI in women in other populations, or whether this variant affects females' BMI trajectory only in the context of the Bangladeshi environmental or genetic background.

The SNV we identified, rs347313, has never been found to be associated with a complex trait phenotype, according to the National Human Genome Research Institute GWAS catalogue. This SNV is situated in the intron of the gene NOS1AP (also known as CAPON) [46], which encodes a cytosolic protein that binds to neuronal nitric oxide synthase, a signaling molecule. NOS1AP has been associated with cardiac phenotypes [47]–[51], and marginally associated with childhood hip circumference in a Hispanic population (p = 8.6·10^−6^ [52]).

Our weight measurement protocol likely captured some intra-individual variability in weight, since participants were interviewed at different times of the day, and during different seasons of the year. However, this variation in the timing of the weight measurement is likely to be smaller than the inter-individual variation in weight, and also not associated with genetics. Therefore, we expect that the noise introduced by this weight measuring protocol would not be systematic, and would not result in confounding between genetics and body mass index. A previous study of BMI and mortality on a subset of the same study population was associated with other clinical endpoints in the expected manner [4]. Therefore we have confidence that the BMI we measured accurately captures the BMI of this population.

In future research, we hope to extend this investigation to examine whether genetics interacts with nutrition in such a way that different variants affect BMI in the presence of different nutrition choices. In the near future, we will have available data on the food intake of these participants, and we intend to investigate the possibility that nutrition mediates the influence of genetic variation on BMI, and the possibility of nutrition interacts with genetic variation to influence BMI.

## Supporting Information

Figure S1 **Manhattan and QQ plots for five traits: (a) body mass index (b) height (c) underweight at baseline (d) overweight at baseline (e) change in BMI over two years.**
(PDF)

Figure S2 **Manhattan and QQ plots for five traits, stratified by gender: (a) BMI in males (b) BMI in females (c) height in males (d) height in females (e) underweight at baseline in males (f) underweight at baseline in females (g) overweight at baseline in males (h) overweight at baseline in females (i) change in BMI over two years in males.** The Manhattan and QQ plots for change in BMI over two years in females are displayed in the main text.
(PDF)

Figure S3 **Manhattan and QQ plots for BMI and change in BMI, stratified by the BMI status of the participant at baseline: (a) BMI in participants who were underweight at baseline (b) BMI in participants who were normal weight at baseline (c) BMI in participants who were overweight at baseline (d) BMI in participants who were underweight at baseline, after removing a single participant with BMI = 51 kg/m^2^ (e) change in BMI over two years in participants who were underweight at baseline (f) change in BMI over two years in participants who were normal weight at baseline (g) change in BMI over two years in participants who were overweight at baseline.**
(PDF)

Table S1 **Socio-demographic and clinical characteristics of the study sample.**
(PDF)

Table S2 **Top associations between SNVs and BMI and change in BMI over two years, stratified by the BMI status of the participant at baseline.**
(PDF)

Table S3 **Heritability estimates for BMI and change in BMI over two years, stratified by the BMI status of the participant at baseline.**
(PDF)
