# Supplementary figures and images for: Genome-Wide Association Studies and Heritability Estimates of Body Mass Index Related Phenotypes in Bangladeshi Adults
Source: PLoS One. 2014 Aug 18;9(8):e105062. doi: 10.1371/journal.pone.0105062 (PMC4136799; doi:10.1371/journal.pone.0105062)

# (a) BMI

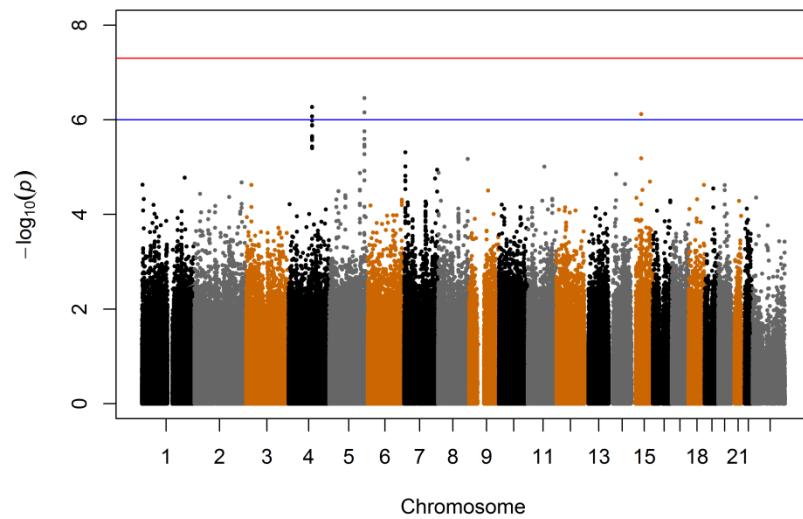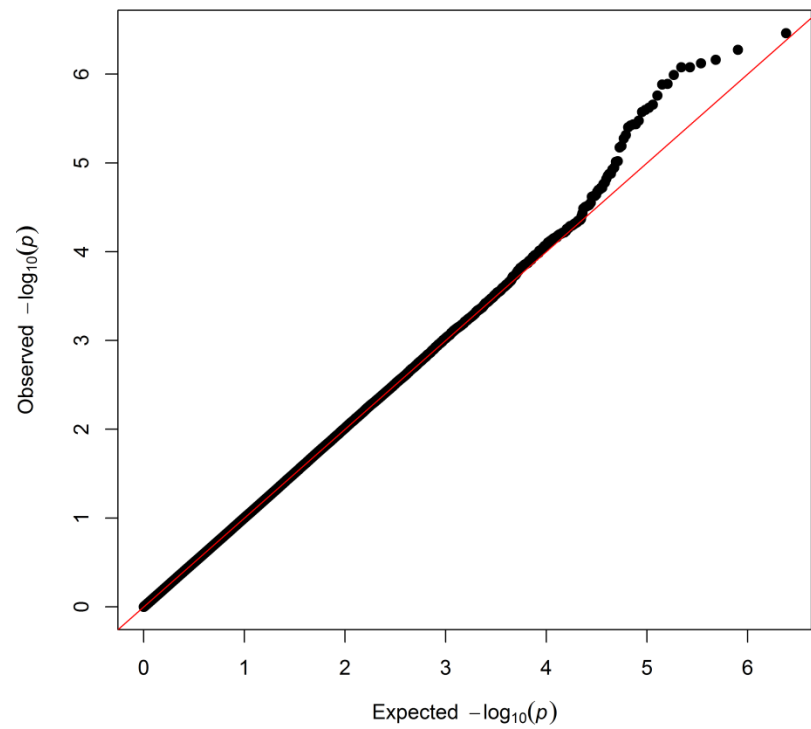

## (b) Height

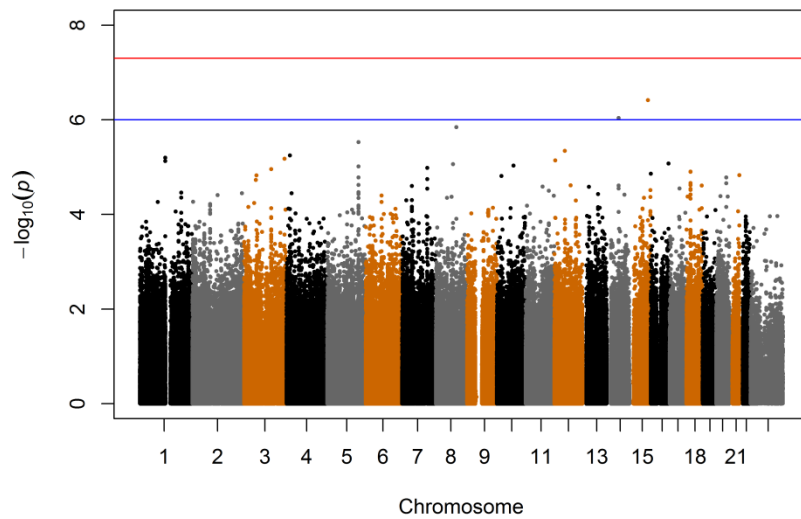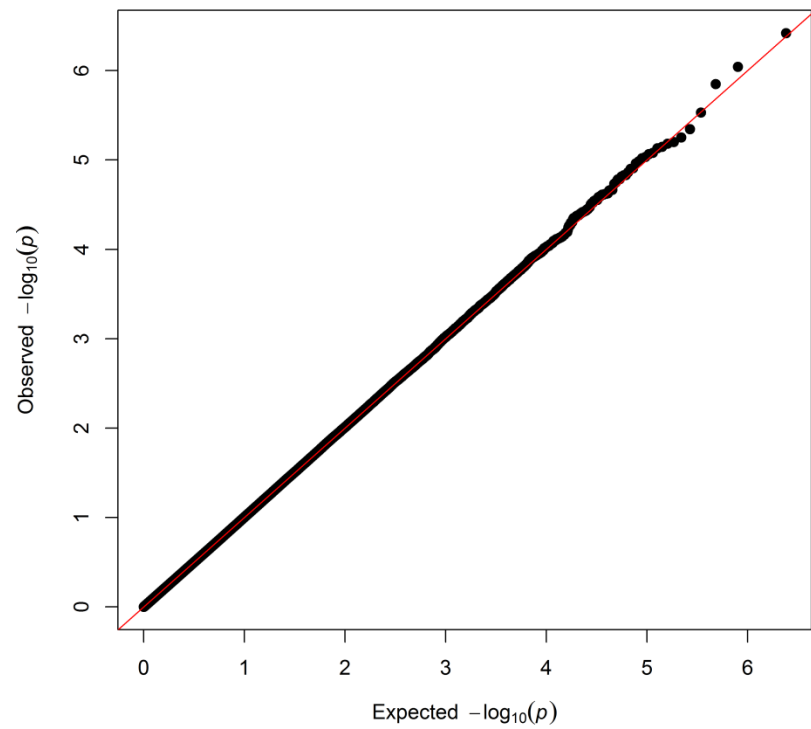

## (c) Underweight at Baseline

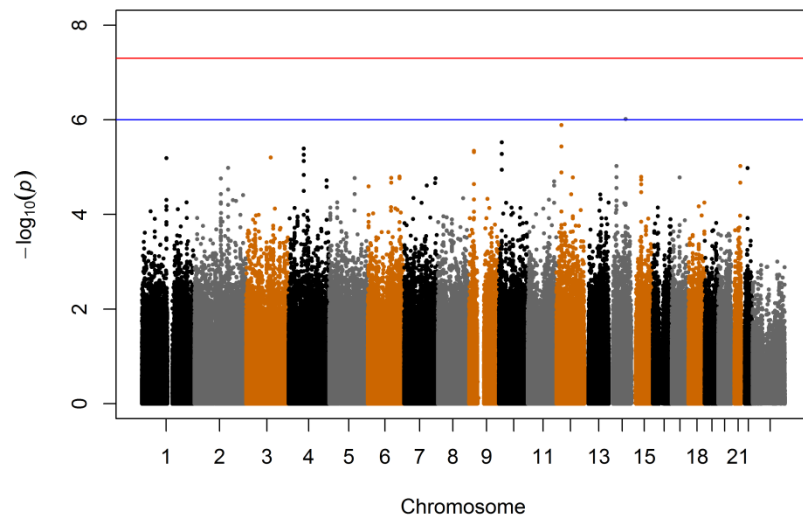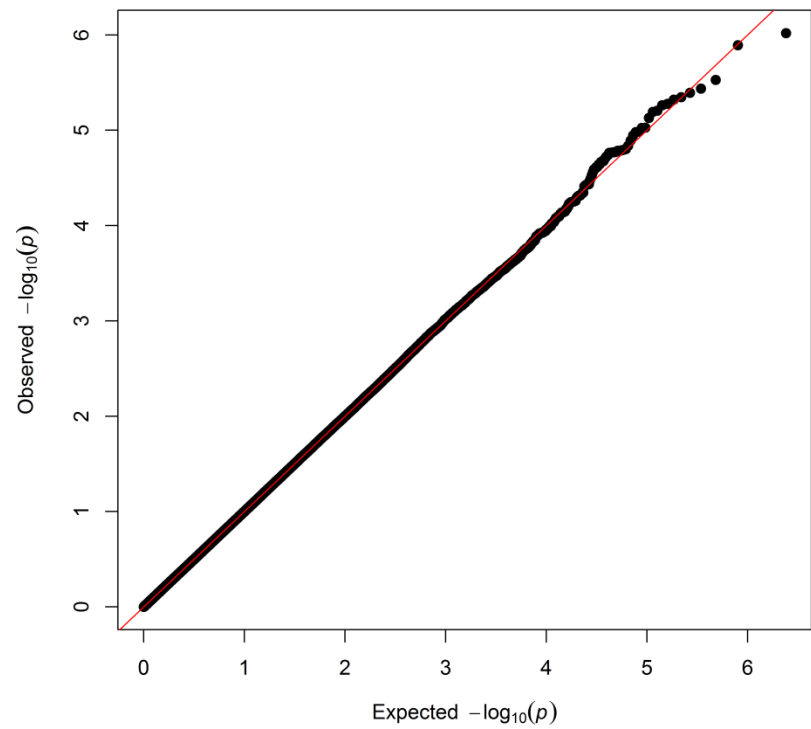

## (d) Overweight at Baseline

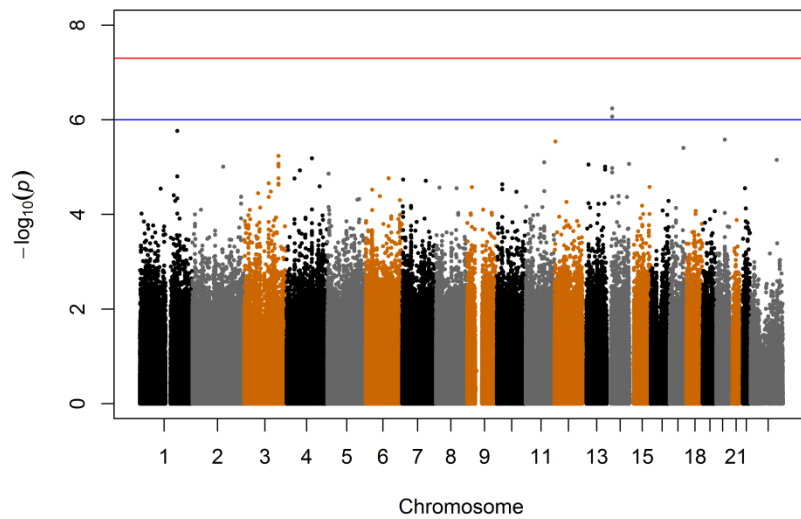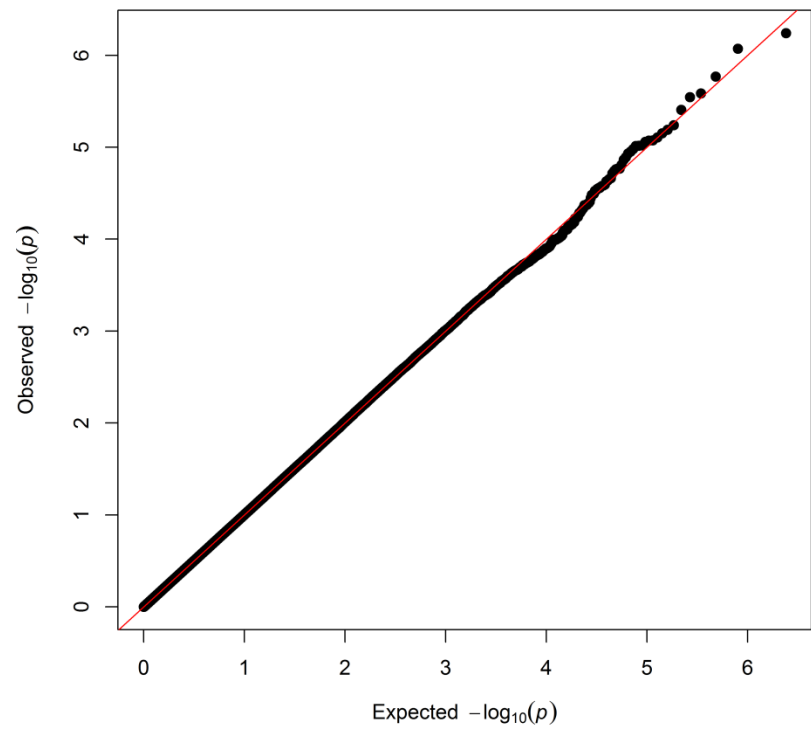

## (e) Change in BMI

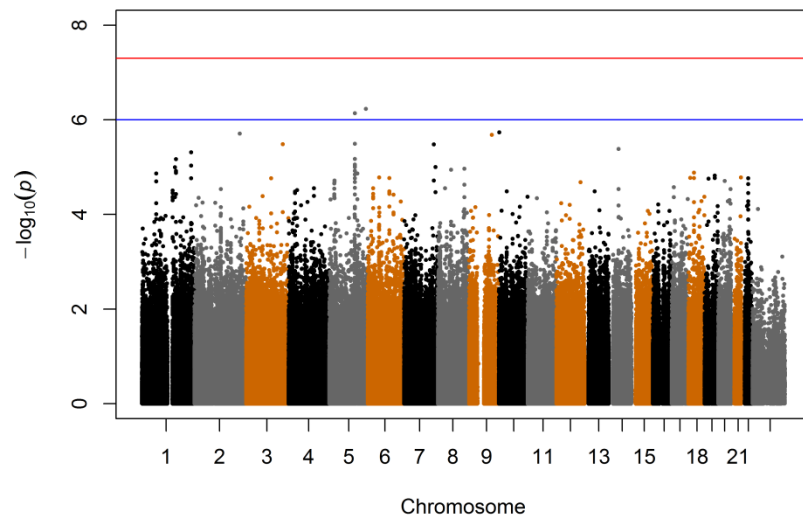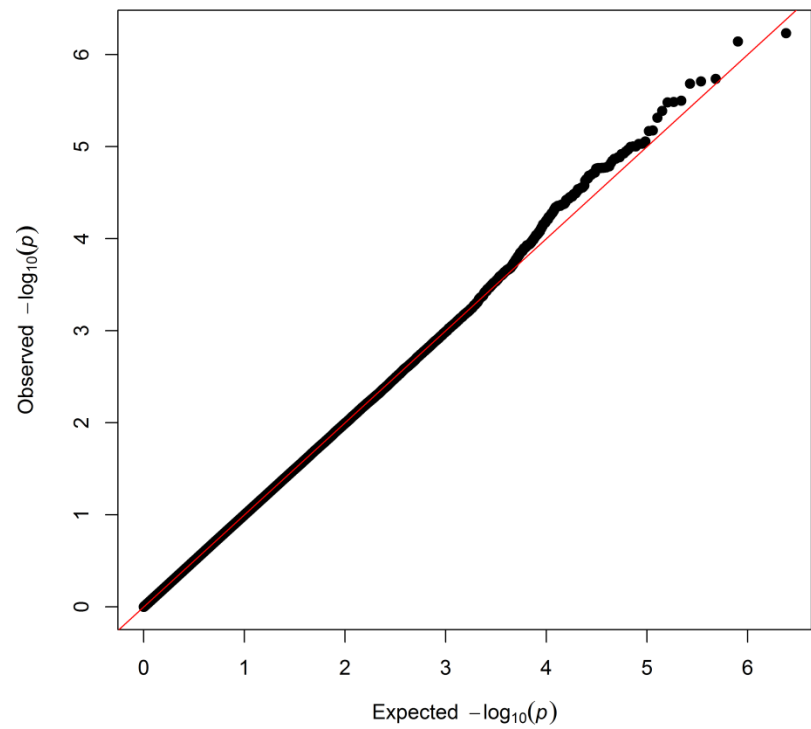

Supplement: Figure S1 — Manhattan and QQ plots for five traits: (a) body mass index (b) height (c) underweight at baseline (d) overweight at baseline (e) change in BMI over two years. (PDF) [file pone.0105062.s001.pdf]
