# Supplementary material for: Genome-Wide Association Studies and Heritability Estimates of Body Mass Index Related Phenotypes in Bangladeshi Adults
Source: PLoS One. 2014 Aug 18;9(8):e105062. doi: 10.1371/journal.pone.0105062 (PMC4136799; doi:10.1371/journal.pone.0105062)

(a) BMI - Males

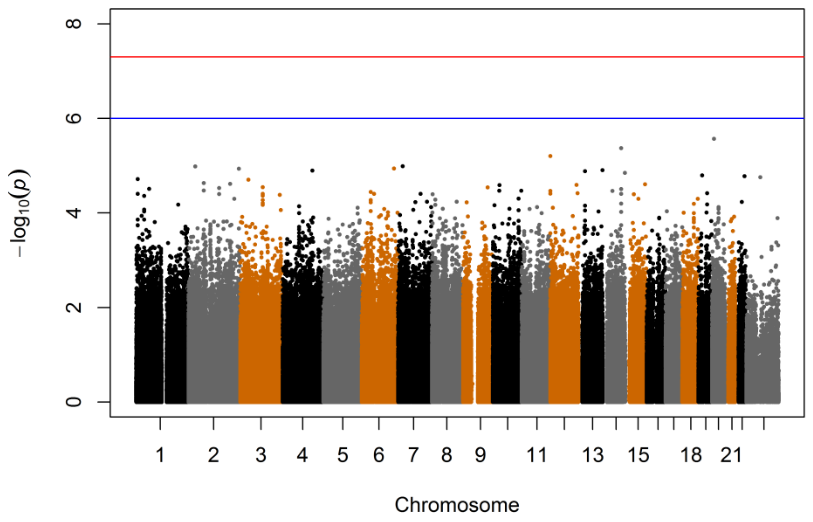

$-\log_{10}(p)$
0
2
4
6
8
1 2 3 4 5 6 7 8 9 11 13 15 18 21
Chromosome

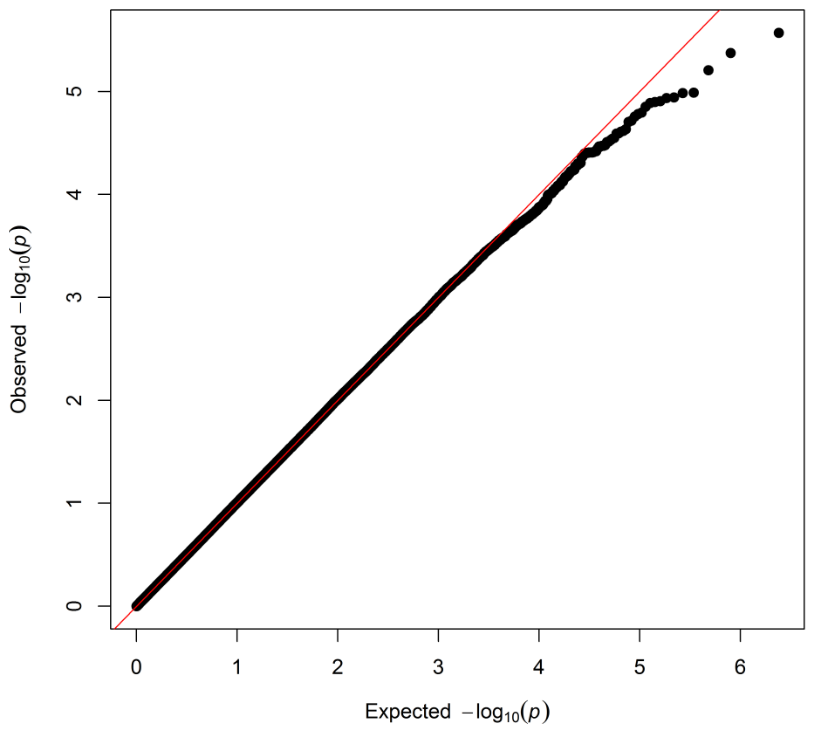

Observed $-\log_{10}(p)$
0
1
2
3
4
5
0 1 2 3 4 5 6
Expected $-\log_{10}(p)$

(b) BMI - Females

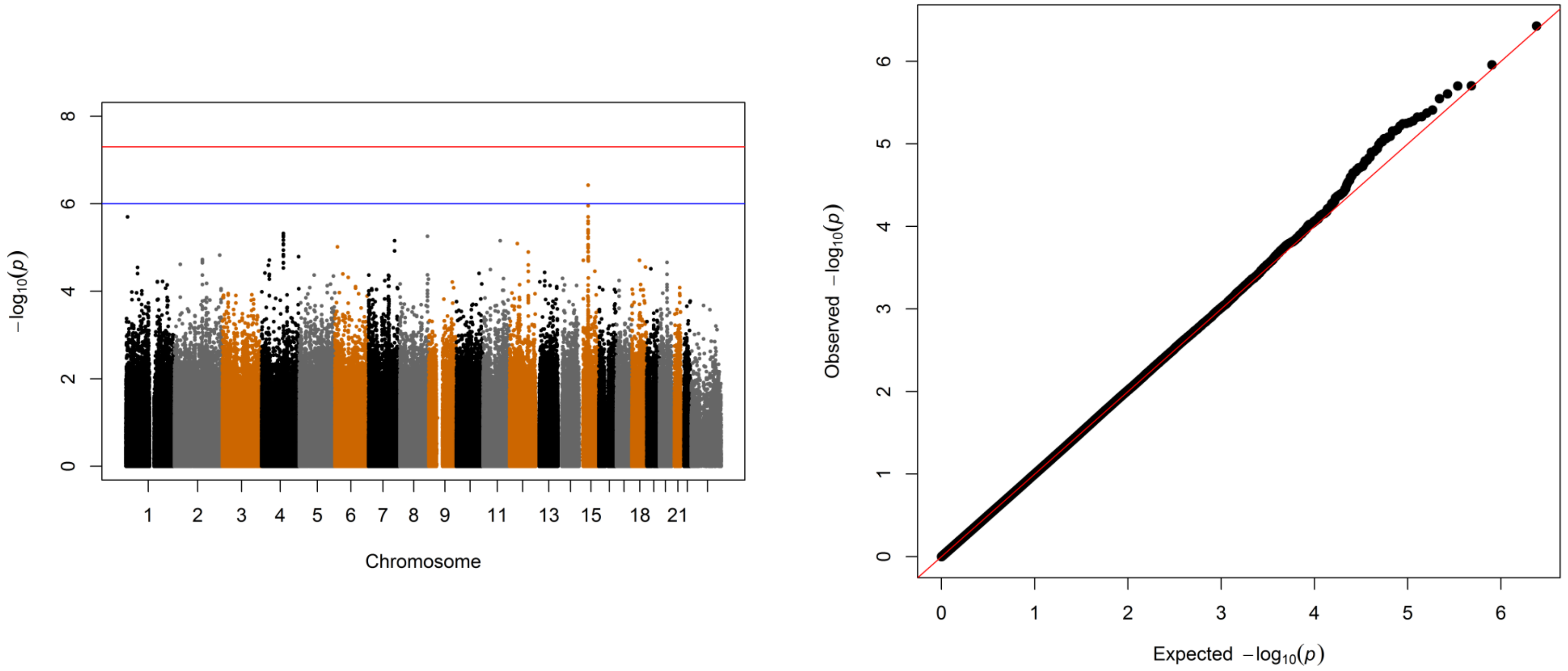

(c) Height - Males

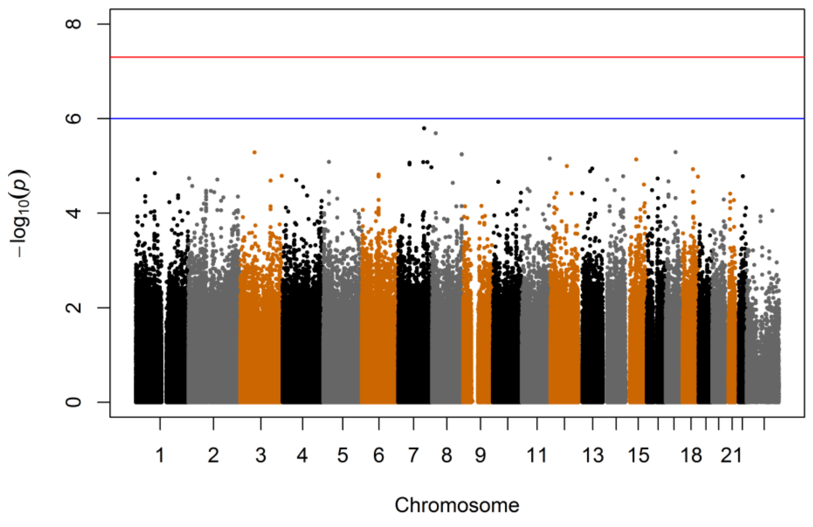

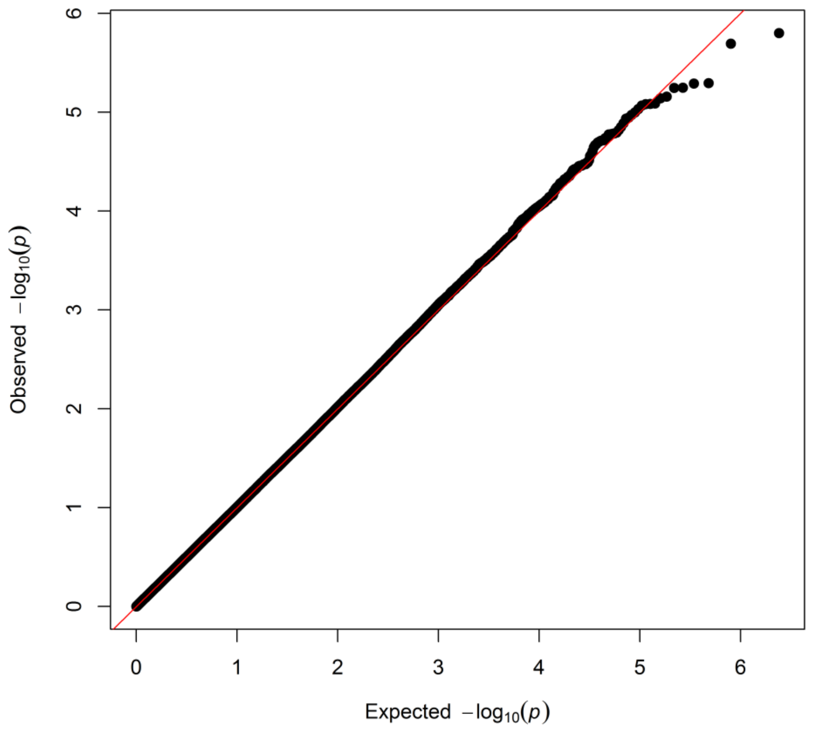

(d) Height - Females

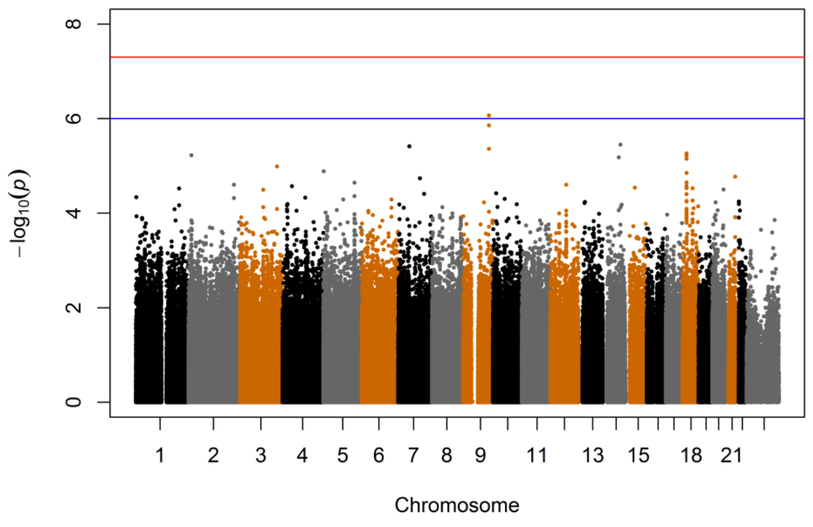

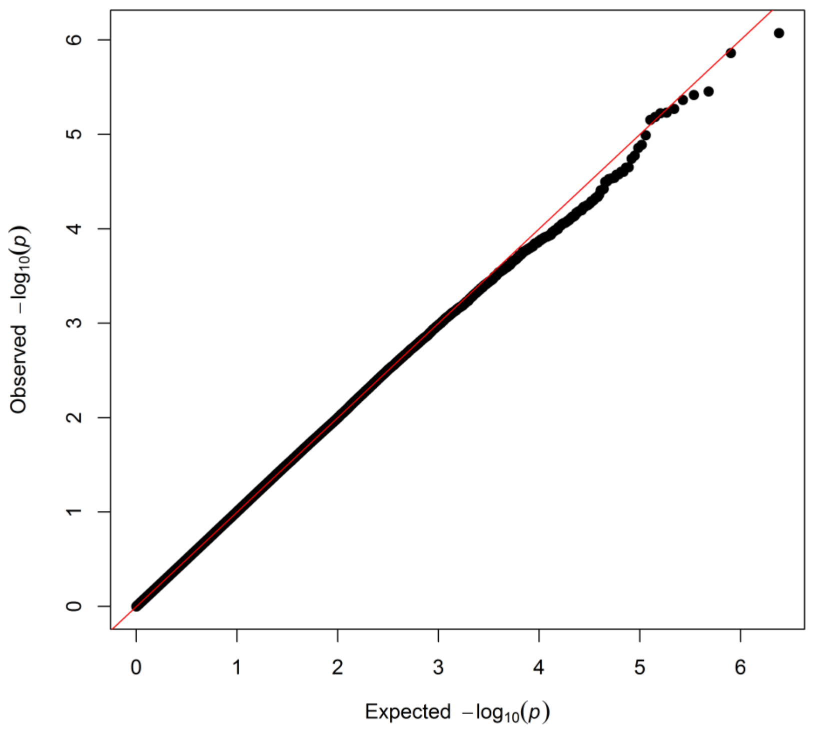

(e) Underweight - Males

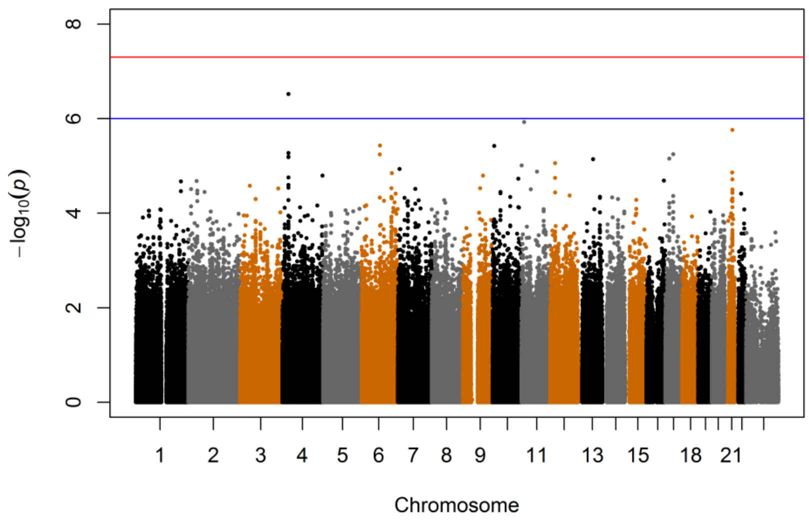

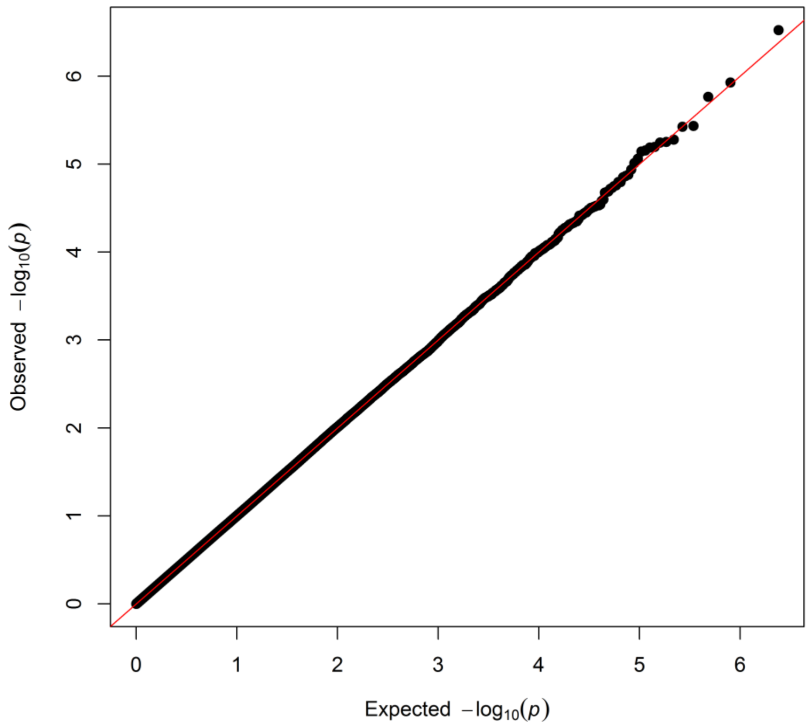

(f) Underweight - Females

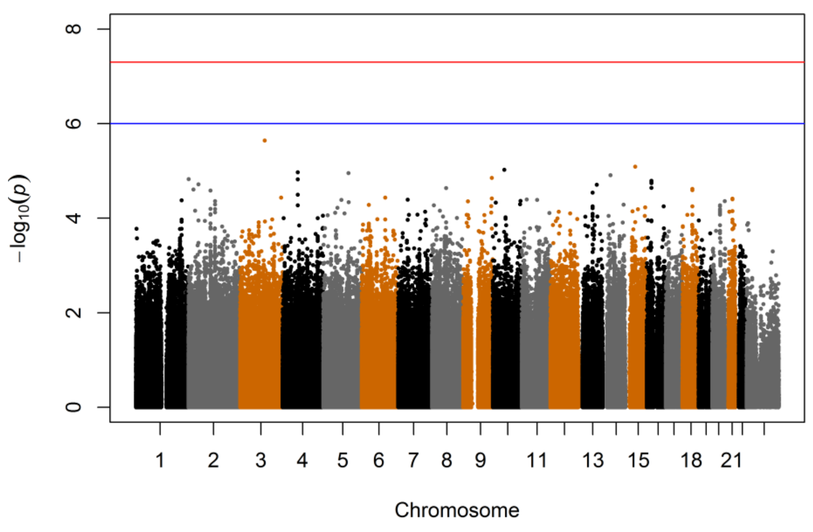

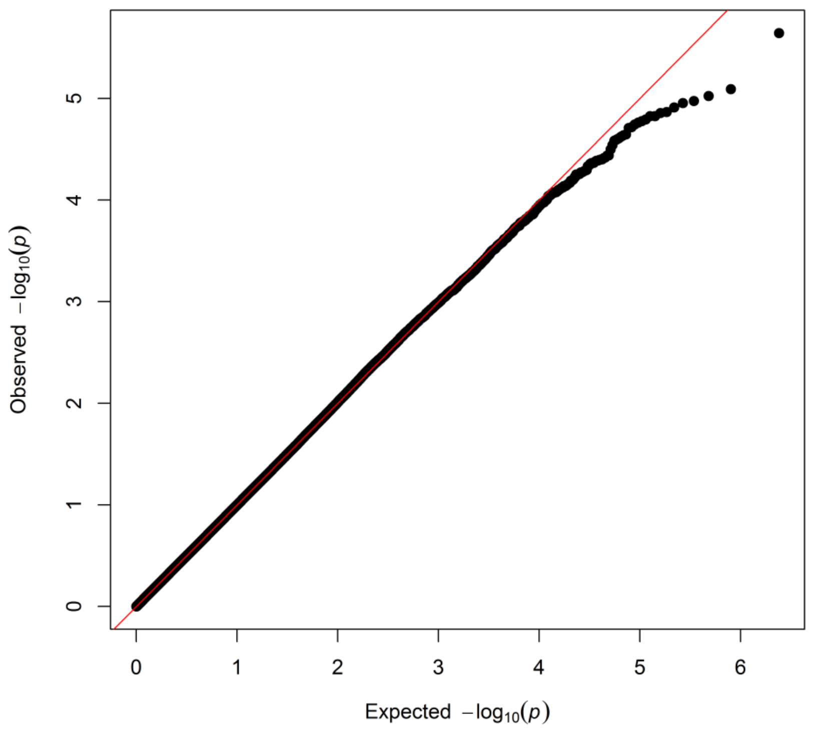

(g) Overweight – Males

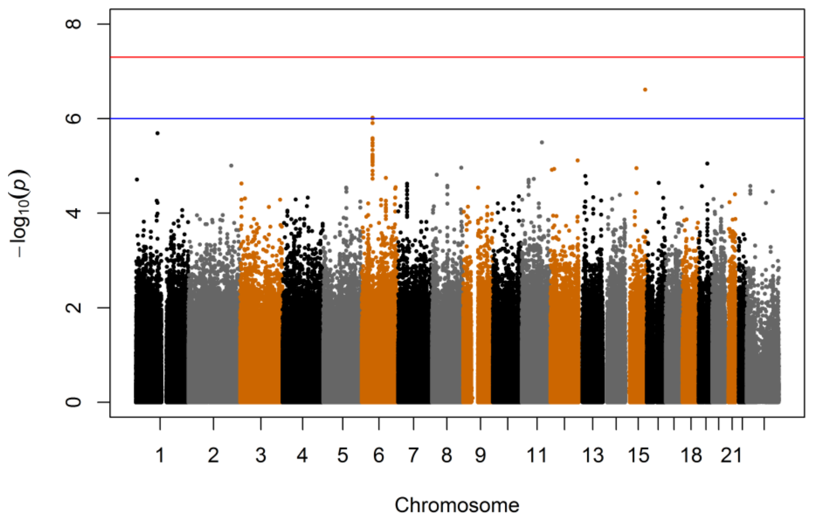

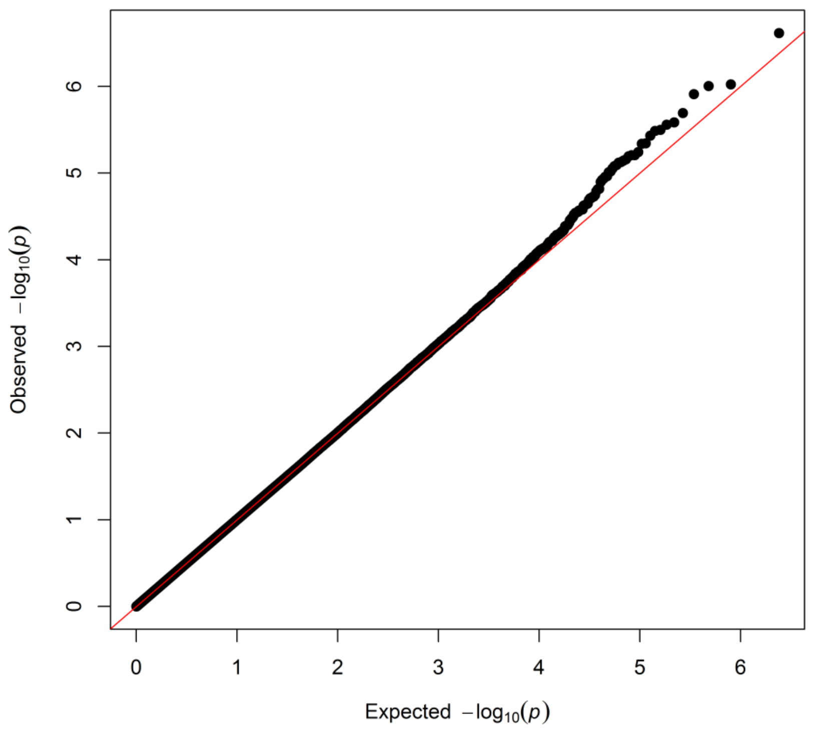

(h) Overweight - Females

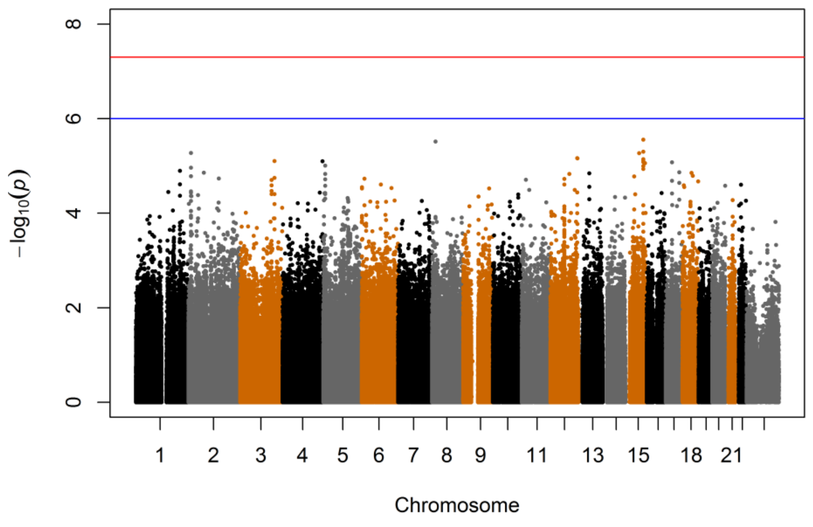

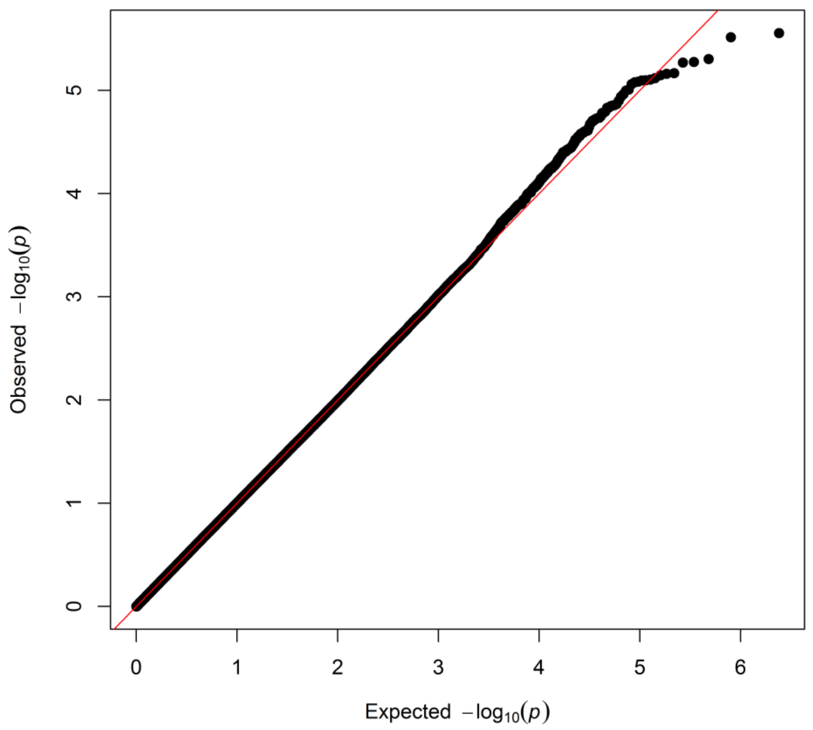

(i) Change in BMI - Males

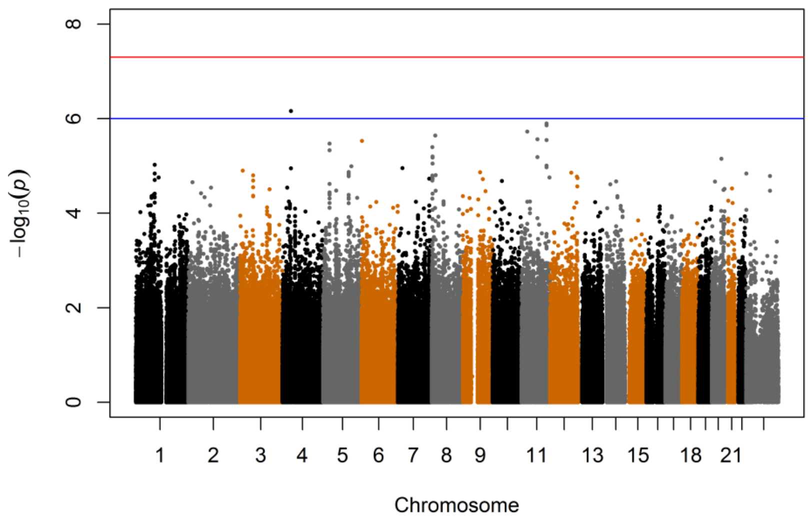

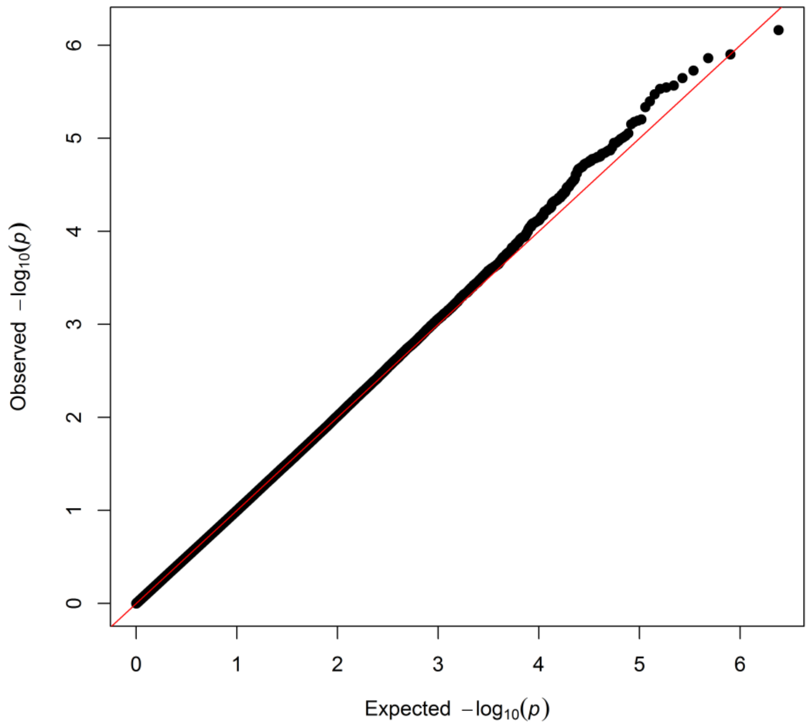

Supplement: Figure S2 — Manhattan and QQ plots for five traits, stratified by gender: (a) BMI in males (b) BMI in females (c) height in males (d) height in females (e) underweight at baseline in males (f) underweight at baseline in females (g) overweight at baseline in males (h) overweight at baseline in females (i) change in BMI over two years in males. The Manhattan and QQ plots for change in BMI over two years in females are displayed in the main text. (PDF) [file pone.0105062.s002.pdf]
