# Supplementary material for: Genome-Wide Association Studies and Heritability Estimates of Body Mass Index Related Phenotypes in Bangladeshi Adults
Source: PLoS One. 2014 Aug 18;9(8):e105062. doi: 10.1371/journal.pone.0105062 (PMC4136799; doi:10.1371/journal.pone.0105062)

(a) BMI – Underweight at Baseline

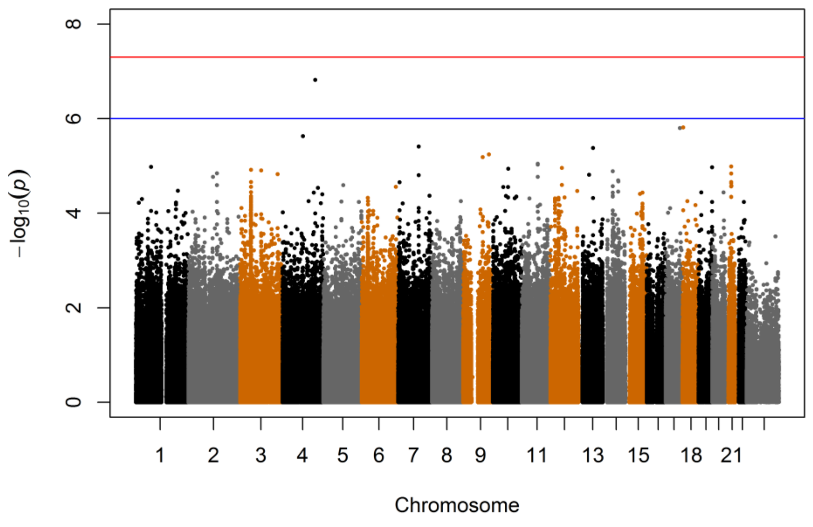

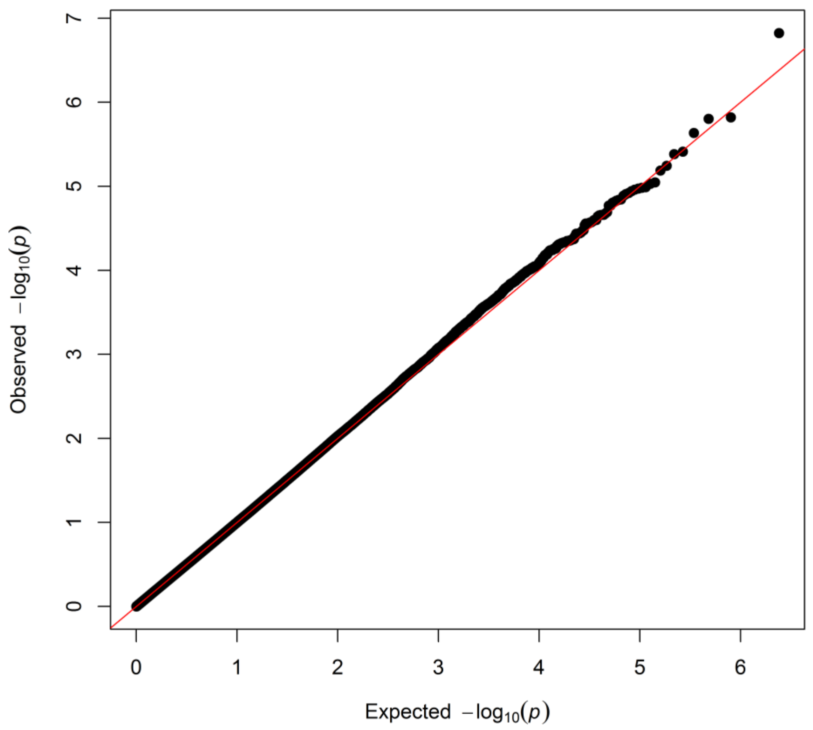

(b) BMI – Normal Weight at Baseline

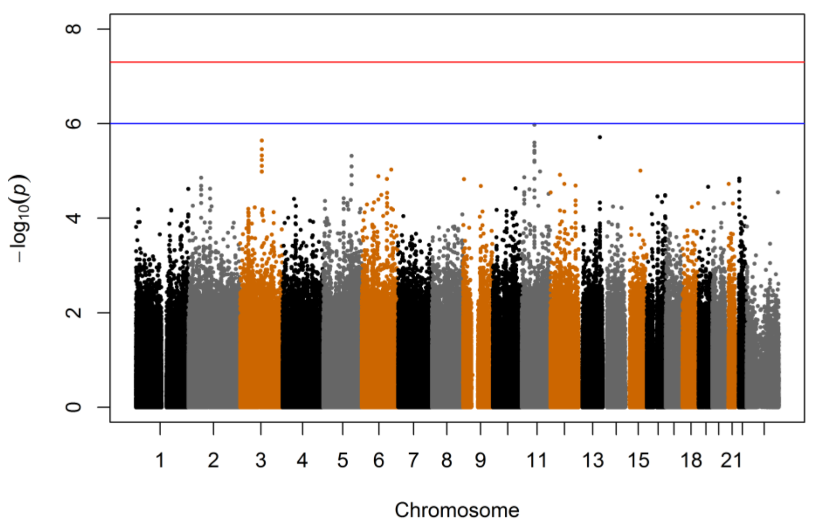

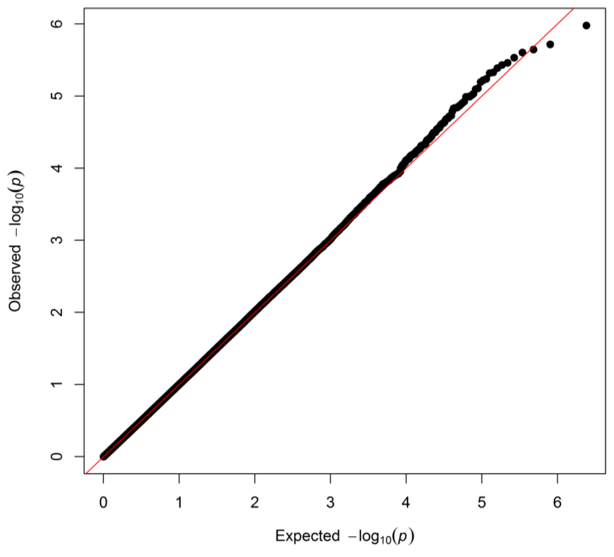

(c) BMI – Overweight at Baseline

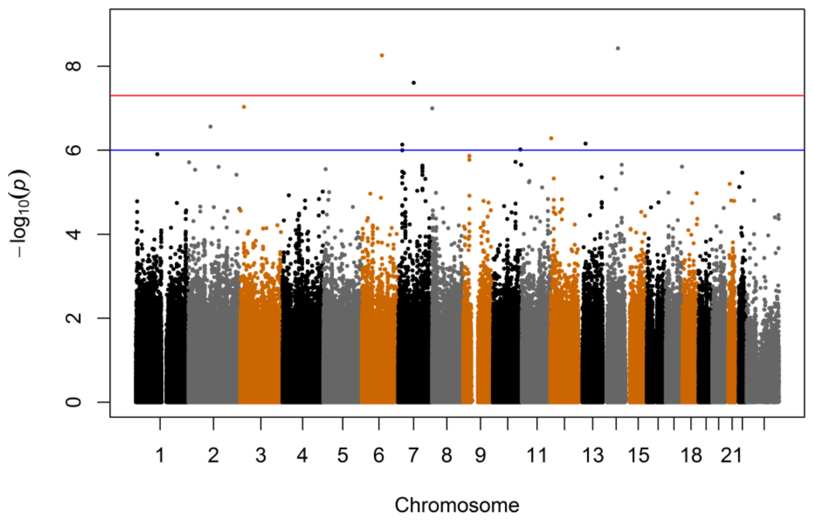

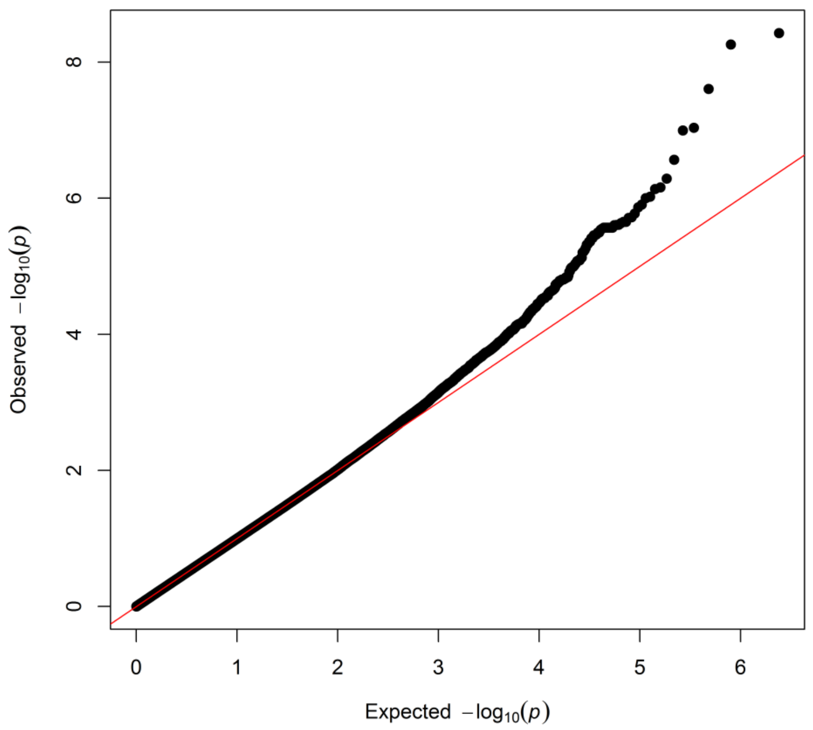

(d) BMI – Overweight at Baseline No Outlier

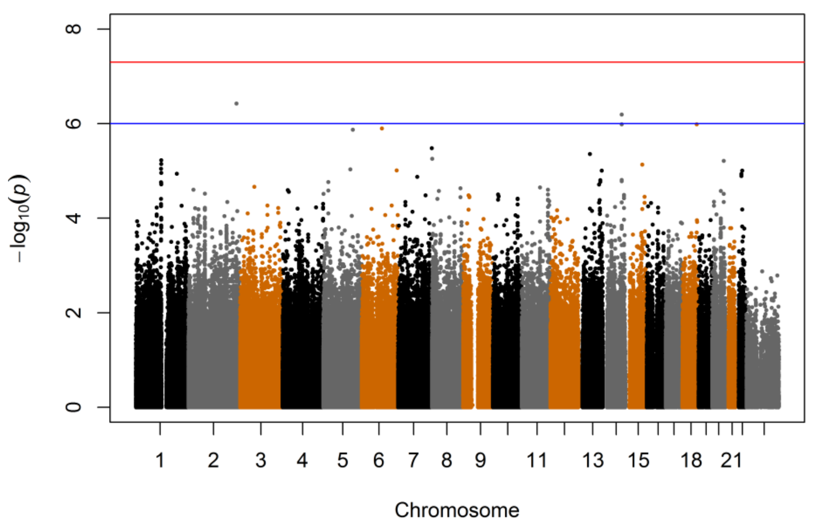

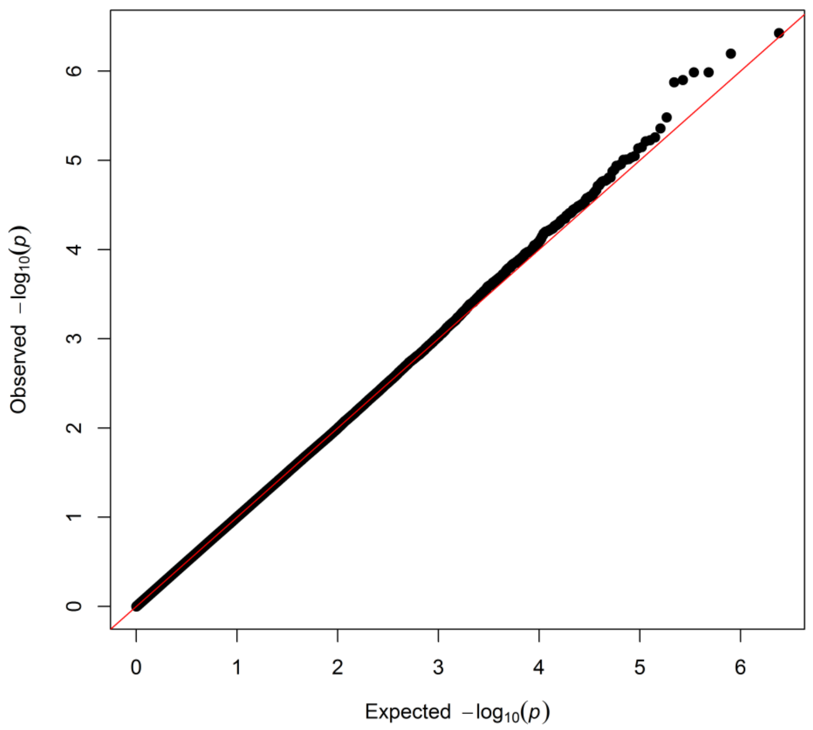

(e) Change in BMI – Underweight at Baseline

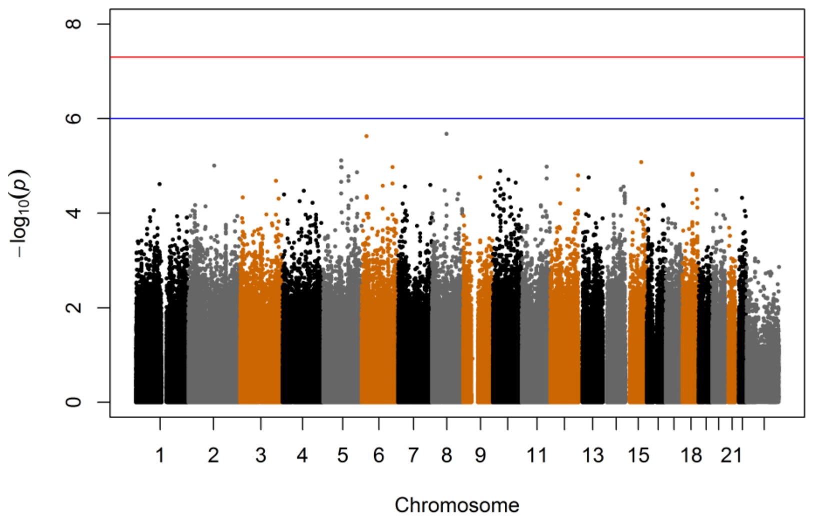

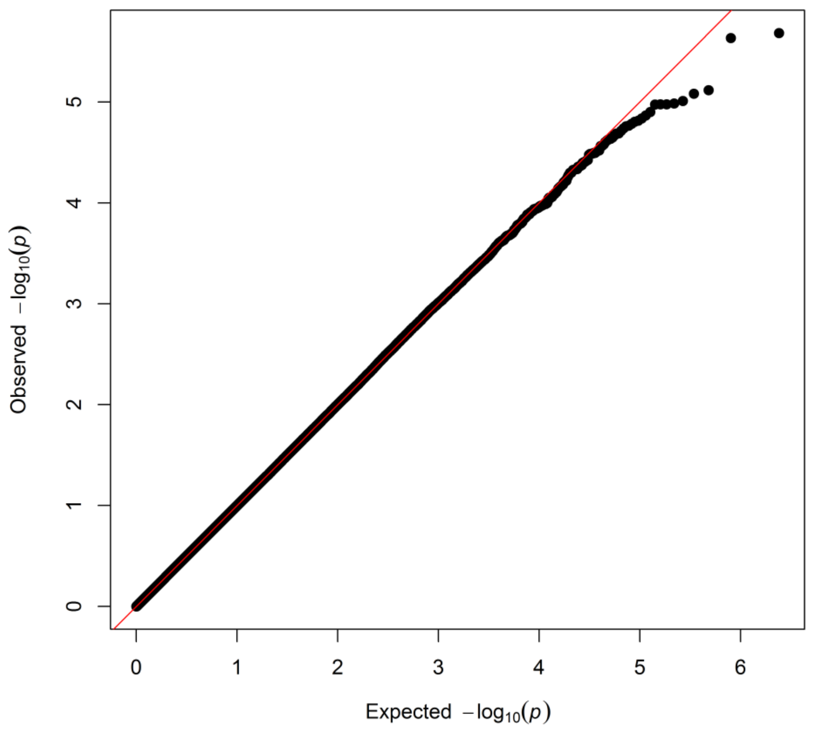

(f) Change in BMI – Normal Weight at Baseline

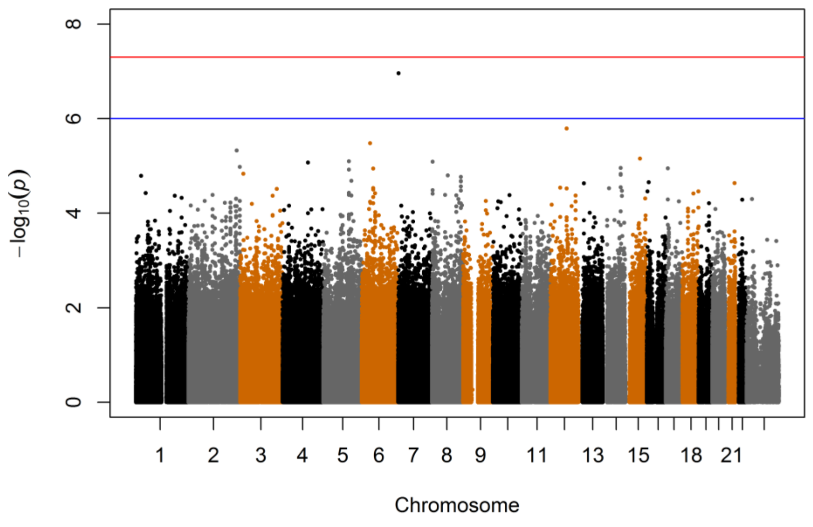

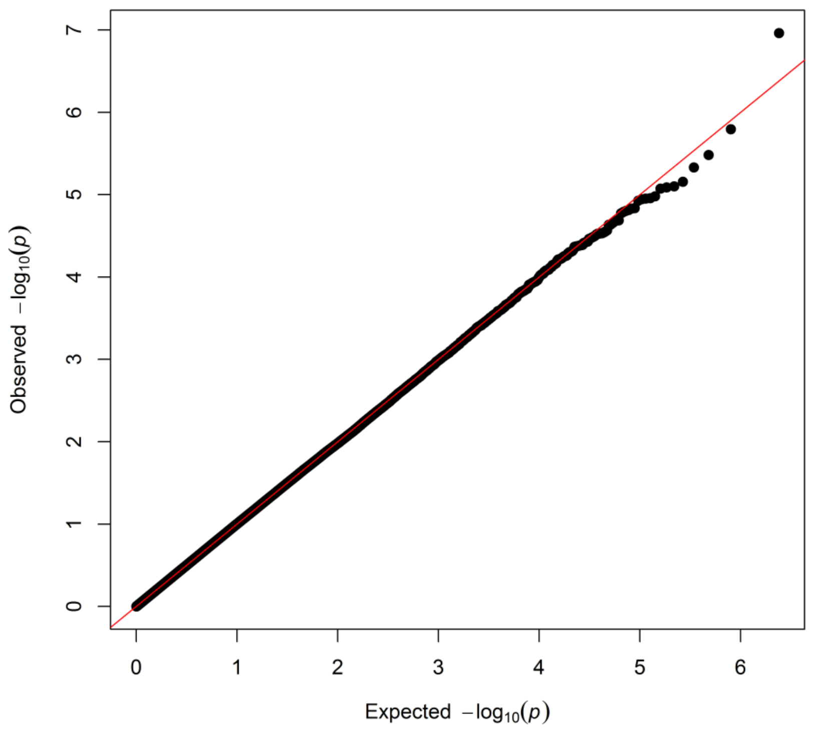

(g) Change in BMI – Overweight at Baseline

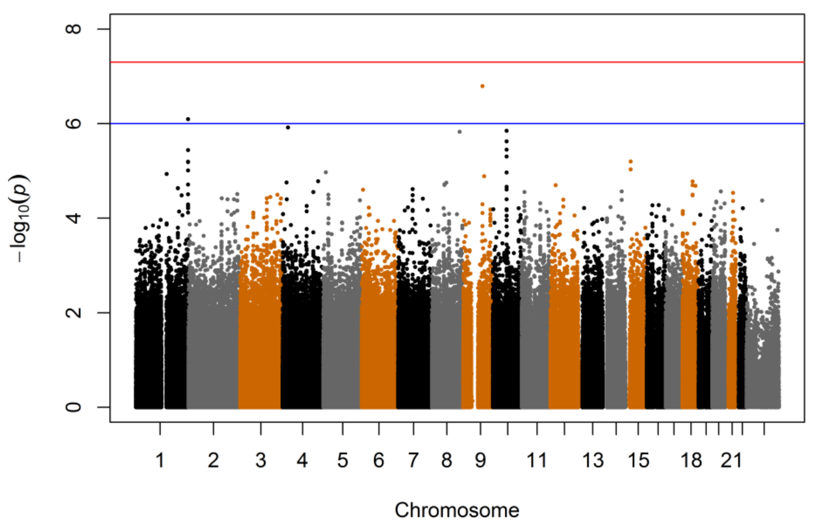

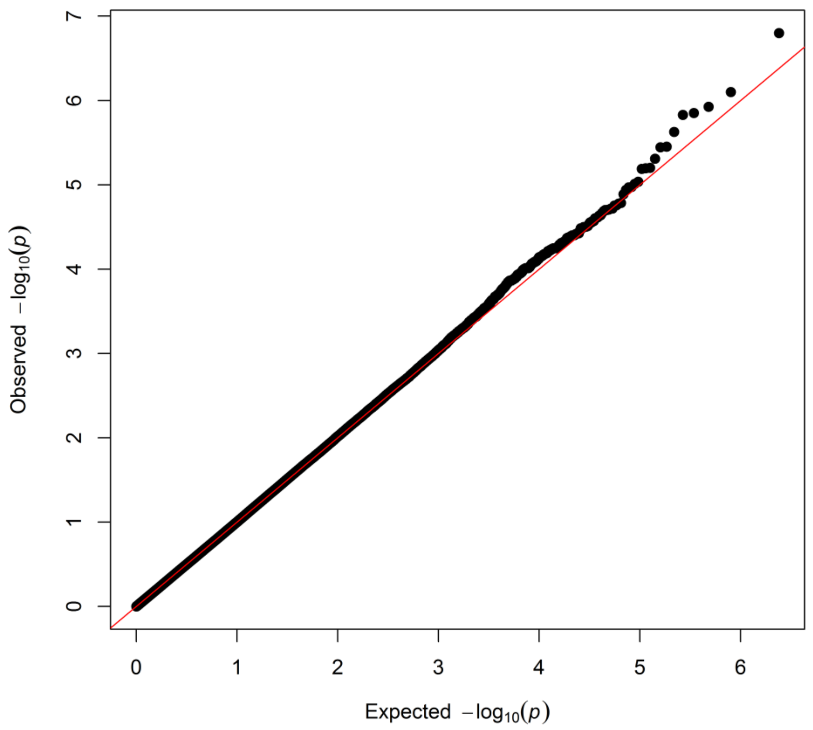

Supplement: Figure S3 — Manhattan and QQ plots for BMI and change in BMI, stratified by the BMI status of the participant at baseline: (a) BMI in participants who were underweight at baseline (b) BMI in participants who were normal weight at baseline (c) BMI in participants who were overweight at baseline (d) BMI in participants who were underweight at baseline, after removing a single participant with BMI = 51 kg/m2 (e) change in BMI over two years in participants who were underweight at baseline (f) change in BMI over two years in participants who were normal weight at baseline (g) change in BMI over two years in participants who were overweight at baseline. (PDF) [file pone.0105062.s003.pdf]
