# Supplementary material for: Genome-Wide Association Studies and Heritability Estimates of Body Mass Index Related Phenotypes in Bangladeshi Adults
Source: PLoS One. 2014 Aug 18;9(8):e105062. doi: 10.1371/journal.pone.0105062 (PMC4136799; doi:10.1371/journal.pone.0105062)
Supplement: Table S1 — Socio-demographic and clinical characteristics of the study sample. (PDF) [file pone.0105062.s004.pdf]

Table S1: Characteristics of the Study Sample

		Combined N=5,354	Men n=2,591	Women n=2,763
Enrolled for HEALS	count (%)	3,364 (62.8)	1,516 (58.5)	1,848 (66.9)
Enrolled for BEST	count (%)	1,990 (37.2)	1,075 (41.5)	915 (33.1)
**Socio-demographic Measurements at Baseline**				
Age (years)	mean (sd)	40 (11.0)	43 (10.9)	37 (10.4)
Married	count (%)	5,019 (93.7)	2,499 (96.5)	2,520 (91.2)
Muslim Religion	count (%)	5,121 (95.7)	2,469 (95.3)	2,652 (96.0)
Owns Land	count (%)	2,610 (48.8)	1,303 (50.3)	1,307 (47.3)
Owns Television	count (%)	2,514 (47.0)	1,227 (47.4)	1,287 (46.6)
Years of Education	mean (sd)	3.4 (4.0)	4.0 (4.2)	2.9 (3.6)
No Formal Education	count (%)	2,446 (45.7)	1,021 (39.4)	1,425 (51.6)
Tobacco: never	count (%)	3,319 (62.0)	754 (29.1)	2,565 (92.8)
Tobacco: former	count (%)	453 (8.5)	366 (14.1)	87 (3.2)
Tobacco: current	count (%)	1,582 (29.6)	1,471 (56.8)	111 (4.0)
Betel: never	count (%)	3,225 (60.2)	1,579 (60.9)	1,646 (65.3)
Betel: former	count (%)	147 (2.8)	102 (3.9)	45 (2.1)
Betel: current	count (%)	1,982 (37.0)	910 (35.1)	1,072 (32.7)
**Clinical Measurements at Baseline**				
Creatinine Adjusted Urinary Arsenic (µg/gm)	mean (sd)	293 (376)	276 (352)	309 (397)
Systolic Blood Pressure (mmHg)	mean (sd)	117 (17.5)	117 (17.2)	117 (17.7)
Diastolic Blood Pressure (mmHg)	mean (sd)	76 (11.0)	76 (10.8)	76 (11.1)
Hypertensive by Blood Pressure	count (%)	819 (15.3)	380 (14.7)	439 (16.0)
Height (m)	mean (sd)	1.55 (0.08)	1.61 (0.06)	1.50 (0.05)
BMI (kg/m^2)	mean (sd)	19.9 (3.3)	19.5 (3.0)	20.2 (3.4)
Underweight	count (%)	2,099 (39.3)	1,146 (44.3)	953 (34.6)
Normal weight	count (%)	2,375 (44.5)	1,106 (42.7)	1,269 (46.1)
Overweight	count (%)	869 (16.3)	338 (13.1)	531 (19.3)
**Clinical Measurements at First Followup**				
BMI (kg/m^2)	mean (sd)	20.1 (3.4)	19.6 (3.1)	20.5 (3.6)
Underweight	count (%)	1,817 (36.5)	968 (41.4)	849 (32.1)
Normal weight	count (%)	2,237 (44.9)	1,045 (44.7)	1,192 (45.1)
Overweight	count (%)	930 (18.7)	327 (14.0)	603 (22.8)

Information is missing for: religion (1 person); land ownership (1 person); TV ownership (1 person); urinary arsenic (33 people); systolic blood pressure (12 people); diastolic blood pressure (15 people); height (10 people); BMI (11 people); and BMI at first followup (103 people were lost due to followup, an additional 263 did not have their BMI recorded). Means and percentages are calculated for participants with non-missing information on that variable.
High blood pressure is defined as measured systolic blood pressure 140 or above, or diastolic blood pressure 90 or above.
BMI is classified as: underweight (BMI < 18.5); normal weight (18.5 <= BMI< 23); or overweight (BMI >= 23).
