# Supplementary material for: Genome-Wide Association Studies and Heritability Estimates of Body Mass Index Related Phenotypes in Bangladeshi Adults
Source: PLoS One. 2014 Aug 18;9(8):e105062. doi: 10.1371/journal.pone.0105062 (PMC4136799; doi:10.1371/journal.pone.0105062)
Supplement: Table S2 — Top associations between SNVs and BMI and change in BMI over two years, stratified by the BMI status of the participant at baseline. (PDF) [file pone.0105062.s005.pdf]

Table S2: Top Associations between SNVs and BMI and change in BMI over two years, by BMI status of the participant at baseline

	subset	n analyzed	Chr	SNV	position	context	nearest gene (s)	RA	AA	RAF	β	se	p-value
**BMI**	Underweight at Baseline	2,099	4	rs17276701	153322015	intergenic	PET112,FBXW7	C	T	0.238	0.219	0.042	1.50E-07
**BMI**	Normal Weight at Baseline	2,375				No variants were associated with a p-value less than 10^{-6}							
**BMI**	Overweight at Baseline	869	14	rs35478231	71209132	intronic	SIPA1L1	A	G	0.012	2.796	0.469	3.73E-09
**BMI**	Overweight at Baseline	869	6	rs370321	95053994	intergenic	TSG1,MANEA	C	A	0.159	0.838	0.142	5.51E-09
**BMI**	Overweight at Baseline	869	7	rs3735505	73453696	intronic	CLIP2	G	A	0.013	2.673	0.475	2.47E-08
**BMI**	Overweight at Baseline	869	3	rs12492931	17925031	intergenic	TBC1D5,LOC339862	T	C	0.054	1.276	0.237	9.20E-08
**BMI**	Overweight at Baseline	869	8	rs7818865	1008657	ncRNA	ERICH1-AS1	T	C	0.051	1.325	0.247	1.01E-07
**BMI**	Overweight at Baseline	869	2	rs11675512	103971651	intergenic	TMEM182,LOC100287010	T	A	0.012	2.531	0.488	2.72E-07
**BMI**	Overweight at Baseline	869	12	rs17700494	4357652	intronic	FGF23	T	G	0.010	2.642	0.522	5.15E-07
**BMI**	Overweight at Baseline	869	13	rs17749200	33405157	intronic	RFC3	C	A	0.036	1.442	0.288	6.92E-07
**BMI**	Overweight at Baseline	869	7	rs12700045	19400327	intergenic	FERD3L,TWISTNB	T	G	0.064	1.067	0.214	7.31E-07
**BMI**	Overweight at Baseline	869	10	rs11597150	128282594	intergenic	C10orf90,DOCK1	A	G	0.013	2.351	0.476	9.52E-07
**BMI**	Overweight at Baseline	869	7	rs4391322	19396112	intergenic	FERD3L,TWISTNB	A	G	0.068	1.024	0.208	9.97E-07
**BMI**	Overweight at Baseline-No Outlier	868	2	rs16866078	225188202	intergenic	CUL3,DOCK10	G	A	0.033	1.404	0.274	3.74E-07
**BMI**	Overweight at Baseline-No Outlier	868	14	rs2896108	88934601	intronic	FOXN3	A	C	0.325	0.527	0.105	6.36E-07
**Change in BMI**	Underweight at Baseline	1,936				No variants were associated with a p-value less than 10^{-6}							
**Change in BMI**	Normal Weight at Baseline	2,228	7	rs34519622	2320868	upstream	SNX8	G	A	0.802	0.273	0.051	9.49E-08
**Change in BMI**	Overweight at Baseline	819	9	rs9410482	91244369	intronic	SEMA4D	C	T	0.926	0.834	0.160	2.23E-07
**Change in BMI**	Overweight at Baseline	819	1	rs11582097	245101654	intronic	AHCTF1	A	G	0.327	0.444	0.089	8.06E-07

AA: Alternative Allele; the allele associated with a decrease in the phenotype; BMI: Body Mass Index; Chr.: Chromosome; Overweight: BMI $\geq$ 23 kg/m^2; compared to normal weight; Position: From build 36.1; RA: Risk allele; the allele associated with an increase in the phenotype; RAF: Risk Allele Frequency; SNV: Single Nucleotide Variant; se: Standard Error; Underweight: BMI < 18.5 kg/m^2, compared to normal weight
