# Supplementary material for: Genome-Wide Association Studies and Heritability Estimates of Body Mass Index Related Phenotypes in Bangladeshi Adults
Source: PLoS One. 2014 Aug 18;9(8):e105062. doi: 10.1371/journal.pone.0105062 (PMC4136799; doi:10.1371/journal.pone.0105062)
Supplement: Table S3 — Heritability estimates for BMI and change in BMI over two years, stratified by the BMI status of the participant at baseline. (PDF) [file pone.0105062.s006.pdf]

Table S3: Heritability Estimates for BMI and change in BMI over two years, stratifying by overweight status of participant at baseline

	Using Whole Population					Excluding related > 0.025		
**Phenotype and Subset**	**n analyzed**	**Estimate of h^2 (a)**	**se**	**Estimate of h_g^2 (b)**	**se**	**n analyzed**	**Estimate of h_g^2 (c)**	**se**
BMI - Underweight at Baseline	2,099	0.227	0.127	0.110	0.098	781	0.000	0.447
BMI - Normal Weight at Baseline	2,375	0.299	0.147	0.274	0.105	955	0.036	0.355
BMI - Overweight at Baseline	869	0.182	0.192	0.284	0.180	389	1.000	0.903
BMI - Overweight at Baseline-no Outlier	868	0.333	0.294	0.480	0.232	389	1.000	0.903
Change in BMI - Underweight at Baseline	1,936	0.375	0.175	0.189	0.127	717	0.000	0.508
Change in BMI - Normal Weight at Baseline	2,228	0.334	0.171	0.341	0.117	899	0.768	0.395
Change in BMI - Overweight at Baseline	819	0.191	0.293	0.352	0.238	365	0.000	0.902

se: standard error

(a) estimate of the full narrow sense heritability, calculated using GCTA, but replacing the full related matrix with a modified one that assumes zero relatedness between participants whose estimated relatedness is less than 0.05

(b) estimate of the amount of variance in the trait that was explained by the interrogated SNVs in a linear model, using the relatedness matrix calculated by GCTA, and using all participants

(c) estimate of the amount of variance in the trait that was explained by the interrogated SNVs in a linear model, using the relatedness matrix calculated by GCTA, and including only one participant of any pair where the estimated kinship coefficient was larger than 0.025 (2nd-3rd cousins)
